# AAO Template-Assisted Fabrication of Vertically Ordered Triangular Pt/Ni Nanopillar Arrays for Enhanced Hydrogen Evolution Reaction

**DOI:** 10.3390/nano16211395

**Published:** 2026-10-30

**Authors:** Zhiqiang Zeng, Mingshu Zheng, Lin Yang, Rui Xu

**Affiliations:** 1 National Laboratory of Solid State Microstructures, School of Physics, Collaborative Innovation Center of Advanced Microstructures, Jiangsu Physical Science Research Center, Nanjing University, Nanjing 210093, China; 2 School of Food Science and Pharmaceutical Engineering, Nanjing Normal University, Nanjing 210023, China; 3 Academician Expert Workstation, Jinhua University of Vocational Technology, Jinhua 321017, China; 4 Institute of Ion Beam Physics and Materials Research, Helmholtz-Zentrum Dresden-Rossendorf e.V. (HZDR), 01328 Dresden, Germany

**Keywords:** Pt/Ni core–shell nanostructures, edge-induced electrocatalysis, finite element simulation

## Abstract

Vertically ordered nanopillar (NP) arrays with sharp nanoedges are attractive for electrocatalytic hydrogen evolution reaction (HER) because the vertically ordered architecture provides efficient transport pathways and large accessible surface areas, while the sharp nanoedges enhance local electric fields and current densities. However, the fabrication of such vertically ordered nanostructures with sharp nanoedges remains challenging. Here, we report an anodic aluminum oxide (AAO) template-assisted strategy to construct vertically ordered triangular Pt/Ni NP arrays with sharp nanoedges for enhanced HER activity. To demonstrate the advantages of triangular NPs, circular Pt/Ni NPs with similar dimensions, composition, Pt chemical states and crystalline phases, and core–shell structures were also fabricated by the same AAO template-assisted strategy as a control to investigate the geometric effect. Electrochemical measurements demonstrate that the triangular NPs exhibit 35% higher HER activity at −100 mV vs. RHE compared to their circular counterparts. Furthermore, COMSOL (Multiphysics 5.5) simulations reveal that the sharp nanoedges induce locally enhanced electric fields and current densities, providing critical insight into the geometry-dependent electrochemical enhancement. This work demonstrates that our AAO template-assisted strategy can construct vertically ordered nanostructures with sharp nanoedges, and that these vertically ordered sharp nanoedges can enhance HER catalytic activity.

## 1. Introduction

Alkaline water electrolysis is one of the most mature industrial technologies for green hydrogen generation [1,2,3,4,5]; the development of highly efficient electrocatalysts remains very important for reducing the energy consumption and improving the overall efficiency of hydrogen generation [1,2,6]. In particular, rational control of catalyst structure provides an effective route to improve electrocatalytic performance beyond conventional compositional optimization. Vertically ordered nanopillar (NP) arrays with sharp nanoedges are particularly attractive for this purpose because their high-curvature edges can generate localized electric-field enhancement and modulate the electrode/electrolyte interfacial environment, potentially promoting interfacial charge transfer and reaction kinetics [7,8,9]. Meanwhile, the vertically ordered architecture provides efficient transport pathways and large accessible surface areas [10,11].

However, fabricating vertically ordered NP arrays with well-defined sharp nanoedges remains challenging [7,12,13]. The key difficulty lies not merely in creating sharp nanoscale features, but in simultaneously achieving vertical ordering, periodic arrangement, and large-area structural uniformity [7,13]. Wet-chemical methods can produce sharp nanostructures, but vertically ordered nanostructure arrays are usually difficult to control over a large area [7,14]. Top-down techniques such as electron-beam lithography and focused ion beam patterning can provide high patterning accuracy, but they are usually expensive, time-consuming, and difficult to scale up for dense three-dimensional nanostructure arrays [15]. More importantly, nanoscale edges have very high curvature and can easily become rounded during lithography, etching, deposition, or post-processing [12]. Bottom-up AAO template-assisted approaches offer a promising alternative owing to their cost-effectiveness, scalability, and ability to replicate designed nanopore geometries [16,17,18]. Our previous studies have demonstrated various AAO-based fabrication strategies, including nanoimprint-assisted anodization methods, enabling the construction of ordered one-dimensional nanostructure arrays over relatively large areas [18]. Nevertheless, producing AAO nanopores with well-defined sharp corners and faithfully replicating their geometry into vertically aligned NP arrays remain difficult. Therefore, despite advances in both top-down and bottom-up fabrication techniques, achieving both well-defined sharp geometry and vertically ordered arrangement over a large area remains a key challenge. In addition, although tip- or edge-induced local field enhancement has been increasingly used to explain improved electrocatalytic activity [7,8,9,11,13], the sharp nanostructures and their reference counterparts in most reported studies are prepared by different synthetic routes and therefore often also differ in composition, crystallinity, exposed facets, catalyst loading, or electrochemically active surface area. Consequently, the intrinsic contribution of nanoedge geometry to the electrocatalytic activity is difficult to isolate, and systematic comparisons on vertically ordered arrays in which geometry is the only deliberately varied parameter remain scarce.

To address these gaps, the objective of this work is to develop an AAO template-assisted strategy for fabricating vertically ordered triangular Pt/Ni NP arrays with sharp nanoedges, and to use these arrays as a well-defined model system to evaluate whether, and to what extent, nanoedge geometry contributes to the alkaline HER activity independently of compositional, crystalline, and surface-area effects. To achieve this objective, the following specific tasks are addressed: (i) to use the highly curved nanocorners of triangular AAO nanopores as nanoconfined templates and to replicate their geometry into Pt-shell/Ni-core NP arrays with uniform dimensions and large-area vertical ordering; (ii) to fabricate circular Pt/Ni NP arrays with closely matched dimensions, Pt loading, and core–shell architecture using the same overall fabrication strategy and identical Pt/Ni deposition conditions as benchmark controls, and to verify by structural and chemical characterization that the two types of arrays differ mainly in their cross-sectional geometry; (iii) to compare the electrochemically active surface area (*ECSA*) normalized HER activity and kinetics of the triangular and circular NP arrays; and (iv) to correlate the measured activity with the local electric-field and current-density distributions obtained from COMSOL simulations. Electrochemical measurements demonstrate that the triangular Pt/Ni NPs exhibit 35% improved HER performance at −100 mV vs. RHE compared with the circular counterparts. COMSOL simulations further reveal pronounced local current-density concentration near the sharp nanoedges of the triangular NPs, supporting a geometry-dependent contribution to the observed electrochemical response. Our study not only provides a practical template-assisted strategy for creating sharp-edged vertical nanostructures, but also demonstrates the potential of nanoscale geometry as a complementary design parameter for electrocatalyst engineering beyond conventional compositional optimization.

## 2. Materials and Methods

### 2.1. Chemicals and Materials

Potassium hydroxide (KOH, ≥99.9%), nickel (II) sulfate hexahydrate (NiSO_4_·6H_2_O, ≥98%), nickel (II) chloride hexahydrate (NiCl_2_·6H_2_O, ≥98%), boric acid (H_3_BO_3_, ≥99.5%), phosphoric acid (H_3_PO_4_, 85%, for analysis EMSURE^®^ACS), and (methylcyclopentadienyl) trimethylplatinum (MeCpPtMe_3_, ≥99.5%) were purchased from Sigma-Aldrich and used as received without further purification.

### 2.2. Fabrication of AAO Templates with Triangular and Circular Nanopores

Triangular-pore AAO templates were fabricated via nanoimprint-assisted anodization. First, electropolished Al foil was patterned using a Ni imprint stamp featuring three-leaf-clover-like nanopillars to create a periodic array of nanoconcaves on the Al surface [19,20]. The nanoimprint process was carried out at 14 MPa for 2 min, where the pressure onto the Ni imprint stamp was applied by a tablet-pressing machine (FW-4A, Tianjin Tianguang New Optical Instrument Technology Co., Ltd., Tianjin, China). Upon completion of imprinting, the Ni imprint stamp was carefully peeled off from the Al foil using tweezers. Afterwards, the pre-patterned Al foil was mounted onto a custom-built anodization cell (Figure S1), where the back side of Al was in good electrical contact with a copper cooling plate connected to the anode terminal. Internal circulating cooling water was circulated through the copper plate to precisely maintain the sample temperature during anodization, and Pt wires were employed as the cathode. Subsequently, anodization was carried out in 0.52 M H_3_PO_4_ aqueous solution at 10 °C under a constant voltage of 140 V supplied by a DC power supply (EA-PS 9000 1U, EA Elektro-Automatik, Viersen, Germany) for 15 min. Under these conditions, the current density was approximately 7.5 mA cm^−2^ in the relatively stable operating region. This process yielded highly ordered triangular nanopores with sharp corners, featuring an average side length of approximately 130 nm, a corner curvature radius of approximately 5 nm, and a template thickness of approximately 600 nm [19]. Circular-pore AAO templates were prepared using a corresponding circular pre-pattern and anodized in the same electrolyte at 10 °C under a constant voltage of 160 V, yielding an average pore diameter of approximately 140 nm and a template thickness of approximately 600 nm [18,21]. After anodization, the templates were rinsed thoroughly with deionized water and dried for subsequent use.

### 2.3. Fabrication of Pt/Ni Nanopillar Arrays

The as-prepared AAO templates with triangular or circular nanopores were subsequently used as nanoconfined templates for the fabrication of Pt/Ni NP arrays. A conformal Pt layer was first deposited onto the inner walls of the AAO nanopores by atomic layer deposition (ALD, Beneq TFS200-106, Beneq Oy, Espoo, Finland) for 300 cycles to form the Pt shell. The ALD process was carried out at 300 °C using (methylcyclopentadienyl) trimethylplatinum (MeCpPtMe_3_) and oxygen as precursors and high-purity Ar (≥99.99%) as the carrier gas. The pulse and purge durations were set to 10 ms and 14 s, respectively. Gravimetric measurement using a micro-analytical balance (METTLER-TOLEDO, XPR2U) was applied to determine Pt areal mass loading via net mass gain of substrates before and after ALD Pt deposition. Three independent replicates were tested for each geometry, yielding average Pt loadings of 63.4 ± 0.6 μg cm^−2^ for triangular NPs and 63.9 ± 0.7 μg cm^−2^ for circular NPs. Subsequently, Ni was electrodeposited into the remaining pore volume to construct Pt-shell/Ni-core NP arrays. The Ni electrodeposition was carried out in a two-electrode configuration using a BioLogic SP-200 potentiostat as the power supply to deliver galvanostatic current. The sample assembled on a custom-designed sample holder served as the working (cathode) electrode. This sample holder was integrated with circulating cooling water to regulate the sample temperature during deposition, while a Pt mesh was used as the counter (anode) electrode. Ni electrodeposition was performed by chronopotentiometry (Figure S12) in 0.8 M NiSO_4_·6H_2_O + 0.2 M NiCl_2_·6H_2_O + 0.4 M H_3_BO_3_ aqueous solution at −2 mA cm^−2^ for 40 min. After deposition, the residual Al and AAO template were removed using aqueous CuCl_2_ solution for Al removal, followed by 5% H_3_PO_4_ for AAO dissolution at 45 °C, releasing vertically ordered Pt/Ni NP arrays on the Ni substrate. Because the Pt layer was formed on the pore walls before Ni filling, the resulting NPs exhibited a Pt-shell/Ni-core configuration. The same fabrication procedure was used for both triangular and circular AAO templates, producing triangular and circular Pt/Ni NP arrays, respectively. The obtained vertically ordered Pt/Ni NP arrays possess continuous ordered domains with lateral size up to 1 cm.

### 2.4. Structural and Chemical Characterization

The morphology and structural arrangement of the Pt/Ni NP arrays and AAO templates were characterized by scanning electron microscopy (SEM; ZEISS GeminiSEM 500, Carl Zeiss, Oberkochen, Germany) operated at an accelerating voltage of 2 kV. The internal structure and elemental distribution of individual Pt/Ni NPs were further investigated by scanning transmission electron microscopy (STEM; Talos F200X, Thermo Fisher Scientific, Eindhoven, The Netherlands) equipped with an energy-dispersive X-ray spectroscopy detector. STEM imaging and EDX elemental mapping were performed at an accelerating voltage of 200 kV. The surface chemical states of Pt were analyzed by X-ray photoelectron spectroscopy (XPS) using a SPECS EnviroESCA system equipped with a one-dimensional delay line detector (1D-DLD). Monochromatic Al Kα radiation (hν = 1486.6 eV) was employed as the excitation source. High-resolution Pt 4f spectra were collected to analyze the chemical states of Pt species. The binding energies were calibrated using the C 1s peak at 284.8 eV, corresponding to the sp^3^ adventitious carbon, to compensate for possible charging effects. The obtained spectra were processed and fitted using XPSPeak 4.1, and the relative fractions of Pt^0^, Pt^2+^, and Pt^4+^ species were determined from the integrated peak areas of the fitted Pt 4f components. X-ray diffraction (XRD; Bruker D8 Advance, Bruker, Karlsruhe, Germany) was employed to probe the crystal structures of the as-prepared NP arrays. Patterns were recorded in Bragg-Brentano (θ–2θ) geometry from 30° to 105° (2θ), using a step width of 0.5°, a step time of 120 s, and Cu Kα radiation (λ = 0.15406 nm).

### 2.5. Electrochemical Measurements

Electrochemical measurements were carried out using a BioLogic SP-200 potentiostat in a conventional three-electrode cell. A reversible hydrogen electrode (RHE; Gaskatel GmbH, Kassel, Germany) and a Pt mesh were used as the reference and counter electrodes, respectively [22,23,24,25,26]. The triangular Pt/Ni NP arrays, circular Pt/Ni NP arrays, bulk Pt and bulk Ni were used as the working electrodes. All HER measurements were performed in H_2_-saturated 1 M KOH at room temperature. Before each measurement, the electrolyte was purged with H_2_ for 30 min to remove dissolved air and ensure H_2_ saturation. The uncompensated solution resistance (R_S_) was determined by electrochemical impedance measurement at a high frequency of 100 kHz, and 85% iR compensation was applied to correct the ohmic potential drop [27].

Cyclic voltammetry (CV) measurements were performed between 0.05 and 1.20 V vs. RHE at a scan rate of 20 mV s^−1^ in 1 M KOH electrolyte saturated with ultrahigh purity N_2_ (≥99.99%) to characterize the electrochemical response of the different electrodes [27]. The electrochemically active surface area (*ECSA*) was determined from the *H_upd_* region of the CV curves. Prior to each measurement, the electrodes were subjected to 50 consecutive CV cycles between 0.05 and 1.20 V vs. RHE to remove surface contaminants and achieve a reproducible Pt surface state. The *H_upd_* charge (*Q_Hupd_*) was obtained by integrating the baseline-corrected current within the selected *H_upd_* potential range (0.05–0.4 V vs. RHE). As illustrated in Figure S14, a baseline (red line) was constructed between the boundaries of the *H_upd_* region and subtracted from the measured current prior to integration. The same potential range and baseline-subtraction procedure were applied to all electrodes to ensure consistent *ECSA* determination. The integrated *H_upd_* charge was converted to *ECSA* using the commonly adopted charge density of 210 μC cm^−2^ for a monolayer of hydrogen adsorbed on Pt:

$$\mathrm{ECSA}=\frac{Q_{H_{\mathrm{upd}}}}{210\mu C\;{\mathrm{cm}}^{-2}}$$

where *Q_Hupd_* is the integrated HUPD charge [28,29]. For CV curves recorded at a 20 mV s^−1^ in 1 M KOH electrolyte, the charge was obtained by integrating the baseline-corrected current with respect to time, *Q_Hupd_* = $\int I_{\mathrm{Hupd}}$ dt. *ECSA* values were derived from at least three independently prepared electrodes per type. Linear sweep voltammetry (LSV) measurements for the HER were conducted at a scan rate of 5 mV s^−1^. The HER current densities were normalized by *ECSA* to minimize the influence of differences in accessible surface area among the electrodes. The overpotential required to reach an *ECSA*-normalized current density of 10 mA cm^−2^ (η_10_) was extracted from the corresponding polarization curves. Tafel slopes were obtained from the linear region of the polarization curves according to the Tafel equation, η = b log (j) + a, where η is the overpotential, j is the current density, and b is the Tafel slope.

### 2.6. COMSOL Multiphysics Simulation

Numerical simulations were performed using the Tertiary Current Distribution, Nernst-Planck interface in COMSOL Multiphysics 5.5 to investigate the geometry-dependent electric field and electrochemical current distribution at the Pt/Ni NP/electrolyte interface. Simplified two-dimensional cross-section triangular and circular NP models were constructed with identical material properties, electrolyte conditions, electrode kinetics, and boundary conditions. It should be highlighted that these simulations are performed in the simplified two-dimensional model, aiming to qualitatively compare geometry-induced local current density and electric field rather than to quantitatively reproduce the absolute experimental current density of three-dimensional nanopillar arrays. The triangular nanopillar was represented by an equilateral Pt triangle with a side length of 130 nm and a finite corner curvature radius of 5 nm, as estimated from the HRTEM characterization of the experimental NPs. For the circular NP, a Pt circle with a diameter of 140 nm was adopted to match the experimental dimension of circular Pt/Ni NPs. The electrolyte was modeled as aqueous 1 M KOH at 298.15 K. Ionic transport included diffusion and migration, whereas convection was neglected. The diffusion coefficients of OH^−^ and K^+^ were set to 5.30 × 10^−9^ m^2^ s^−1^ and 3.75 × 10^−9^ m^2^ s^−1^, respectively, and the relative permittivity of the electrolyte was set to 78.5. The more detailed parameter was summarized in Table S3. Charge transfer at the electrode/electrolyte interface was described using Butler–Volmer kinetics with two transferred electrons and an equilibrium potential of 0 V. The same electrochemical parameters were used for the compared geometries. The electrolyte potential at the outer boundary was fixed at 0 V, while the remaining non-electrode boundaries were treated as electrically insulating and impermeable to ionic flux. The model was solved using a stationary study with an extremely fine mesh at an electrode potential of −50 mV for both geometries, enabling a direct comparison of the current-density and electric field distributions.

## 3. Results and Discussion

### 3.1. Structural Characterization

**Figure 1:**
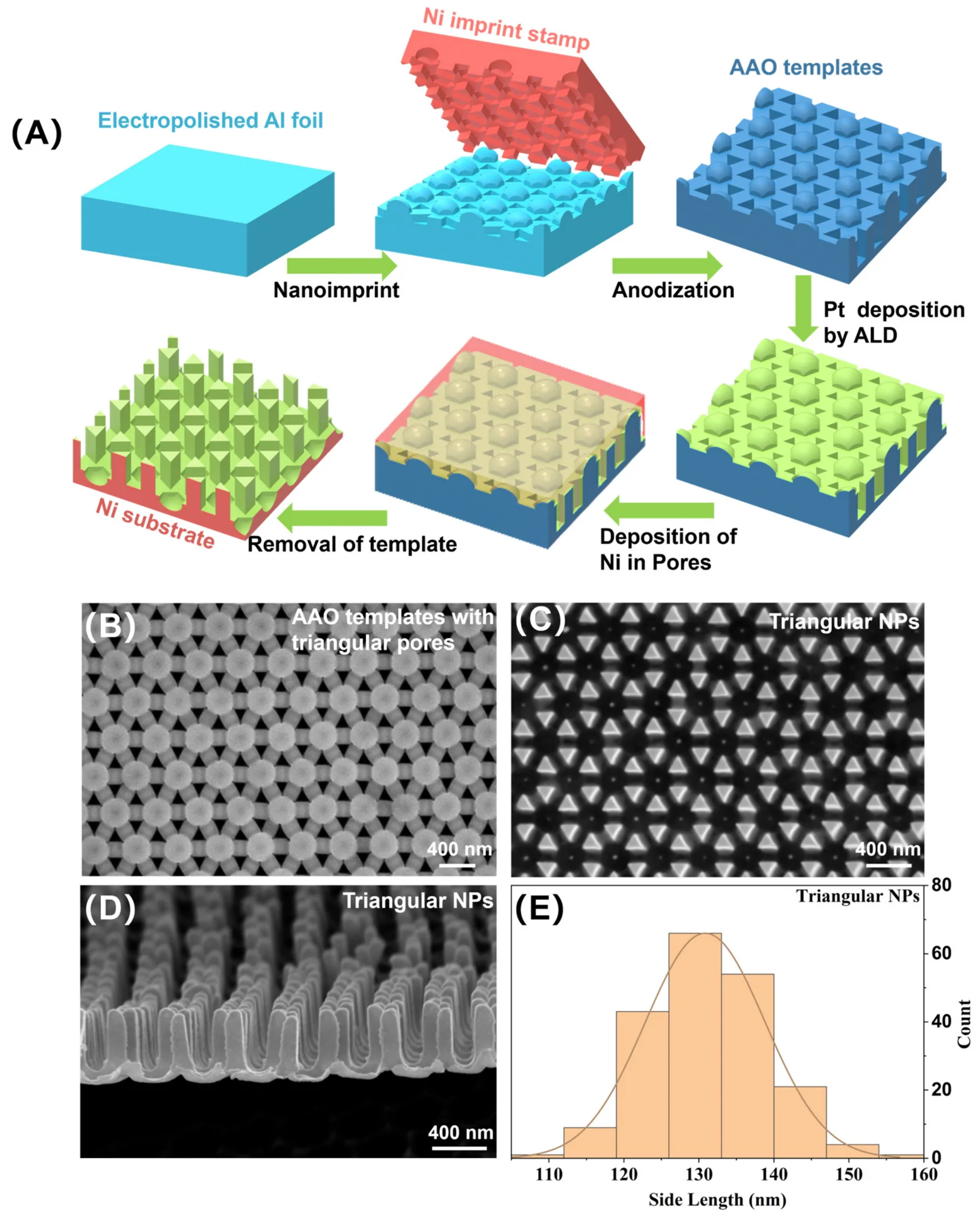
Fabrication and morphological characterization of AAO templates and vertically ordered triangular Pt/Ni nanopillars (NPs). (**A**) Schematic illustration of the AAO-template-assisted fabrication process for triangular NPs. (**B**) Top-view SEM image of the AAO template with triangular nanopores. (**C**,**D**) Top-view and cross-section SEM images of the triangular NPs, respectively, showing their triangular geometry and highly ordered arrangement. (**E**) Statistical distribution of the triangular NP side length, showing an average value of approximately 130 nm.

To construct ordered Pt/Ni NP arrays with sharp nanoedges, we used an AAO-template-assisted method to fabricate triangular Pt/Ni NP arrays, as illustrated in Figure 1A. First, a highly ordered array of triangular nanopores (Figure 1B and Figure S2) was formed in anodic aluminum oxide (AAO) on an Al substrate through nanoimprint patterning followed by anodization. Statistical analysis of the triangular nanopores gives an average side length of approximately 130 nm (Figure S3), indicating good dimensional uniformity of the AAO template. A conformal Pt layer was then deposited onto the inner surfaces of the triangular nanopores by atomic layer deposition (ALD), serving as the Pt shell of the resulting NPs. Subsequently, Ni was electrochemically deposited into the Pt-coated nanopores to form the Ni cores. Finally, the residual Al substrate and AAO template were removed by wet etching, yielding vertically ordered triangular Pt/Ni core-shell NP arrays.

Figure 1C,D show the top-view and cross-section SEM images of the triangular Pt/Ni NP arrays, revealing a highly ordered periodic arrangement with distinct sharp nanoedges. Statistical analysis of the NP dimensions (Figure 1E) gives an average side length of approximately 130 nm, demonstrating good dimensional uniformity. The low-magnification SEM image in Figure S4 further demonstrates the large-area uniformity of the arrays over a field of view spanning approximately 24 μm. STEM-EDX elemental mapping confirms the core-shell configuration of the triangular Pt/Ni NPs (Figure S5). The Ni signal is predominantly confined to the interior, whereas the Pt signal is distributed throughout the outer region and remains detectable at the sharp triangular tips. Importantly, no apparent Ni exposure is observed at the nanoedges, indicating that the Ni core is effectively encapsulated by the Pt shell at the tips. A representative STEM-EDX line profile indicates a Pt shell thickness of approximately 15 nm, further supporting the core-shell structure of the Pt/Ni NPs (Figure S6).

To investigate the effect of sharp nanoedges on the electrochemical performance, circular Pt/Ni NP arrays were fabricated using an AAO template with circular pores (Figure S7) as a control. As shown in Figure S7, the circular NPs also exhibit a highly ordered arrangement and good structural uniformity, but without the sharp nanoedges of the triangular structures. Statistical analysis gives an average diameter of approximately 140 nm (Figure S9), while a representative STEM-EDX line profile indicates a Pt shell thickness of approximately 15 nm (Figure S10). Thus, the two Pt/Ni NP arrays possess closely matched dimensions and Pt shell thicknesses while differing primarily in their geometry, providing a suitable platform for investigating geometry-dependent HER behavior.

**Figure 2:**
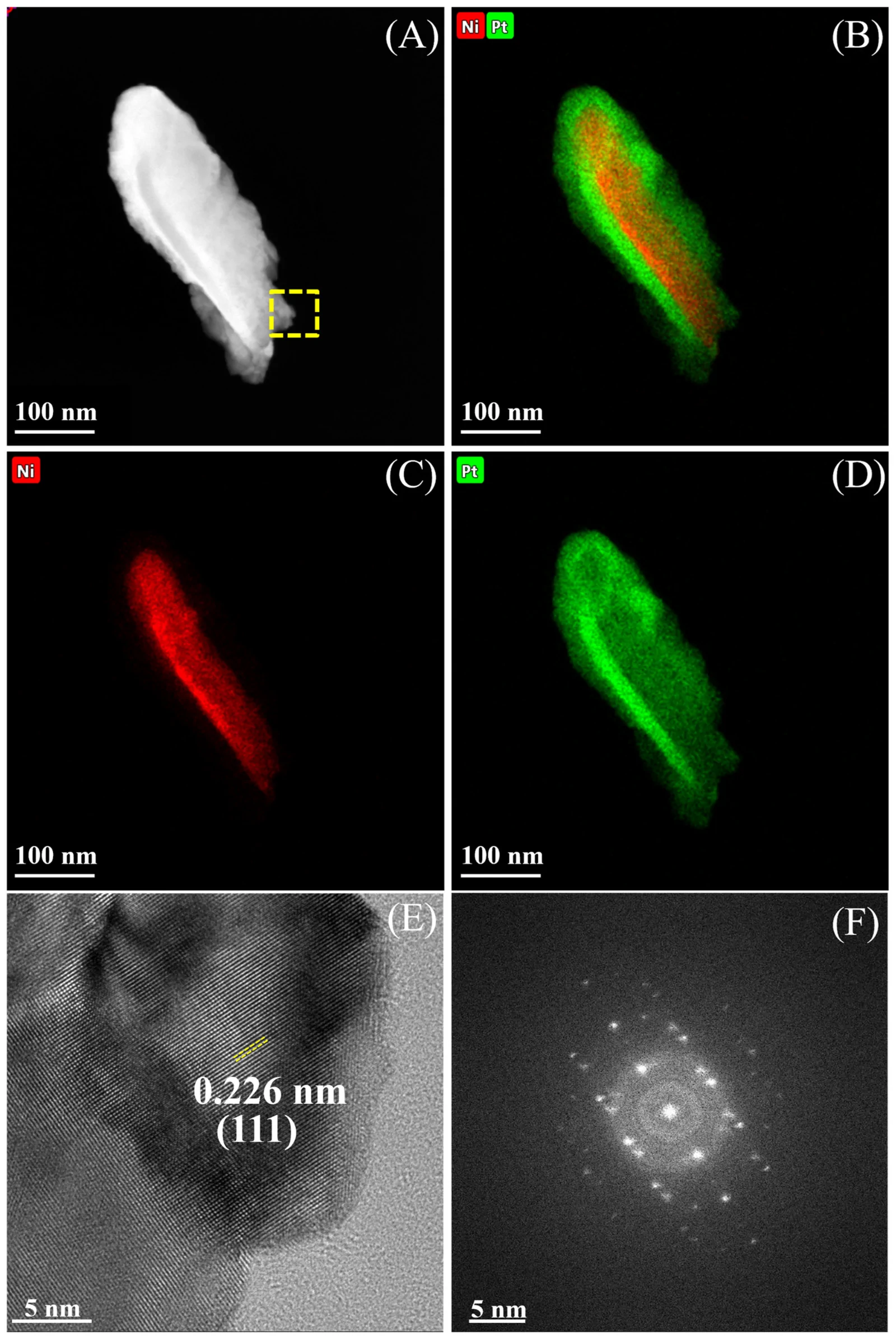
Structural and compositional characterization of triangular Pt/Ni nanopillars. (**A**) STEM image of a triangular Pt/Ni nanopillar, with the yellow dashed box indicating the region selected for high-resolution analysis. (**B**) Overlaid EDX elemental map of Ni and Pt. (**C**,**D**) Individual EDX maps of Ni and Pt, respectively, revealing a Pt-shell/Ni-core elemental distribution. (**E**) HRTEM image acquired from the selected region in (**A**), showing clear lattice fringes with an interplanar spacing of approximately 0.226 nm, consistent with the Pt (111) planes. (**F**) Corresponding FFT pattern confirming the crystalline nature of the Pt-containing shell region.

To further resolve the internal elemental distribution and local crystalline structure of the Pt/Ni NPs, STEM, EDX elemental mapping, HRTEM, and FFT analyses were performed. As shown in Figure 2A, the triangular Pt/Ni NP retains a well-defined triangular morphology with pronounced sharp nanoedges. The overlaid EDX map (Figure 2B) together with the individual Ni and Pt maps (Figure 2C,D) shows that Ni is predominantly distributed in the inner region, whereas Pt is enriched along the outer surface, confirming the Pt-shell/Ni-core configuration. HRTEM analysis of the shell region indicated by the yellow dashed box in Figure 2A reveals well-resolved lattice fringes with an interplanar spacing of approximately 0.226 nm (Figure 2E), consistent with the Pt (111) planes. The corresponding FFT pattern exhibits distinct diffraction features, further confirming the crystalline nature of the Pt-containing shell region (Figure 2F).

For comparison, the circular Pt/Ni NPs exhibit a similar elemental and crystalline structure (Figure S11). EDX mapping likewise reveals Ni-rich inner regions surrounded by a Pt-rich shell, while HRTEM analysis of the outer region shows an interplanar spacing of approximately 0.226 nm, again consistent with Pt (111). The corresponding FFT pattern also confirms the presence of crystalline domains. Notably, the similar Pt (111) lattice spacing observed in both triangular and circular NPs indicates that the change in NP geometry does not introduce an obvious difference in the local Pt lattice structure.

### 3.2. Crystalline Phases and Surface Chemical States

**Figure 3:**
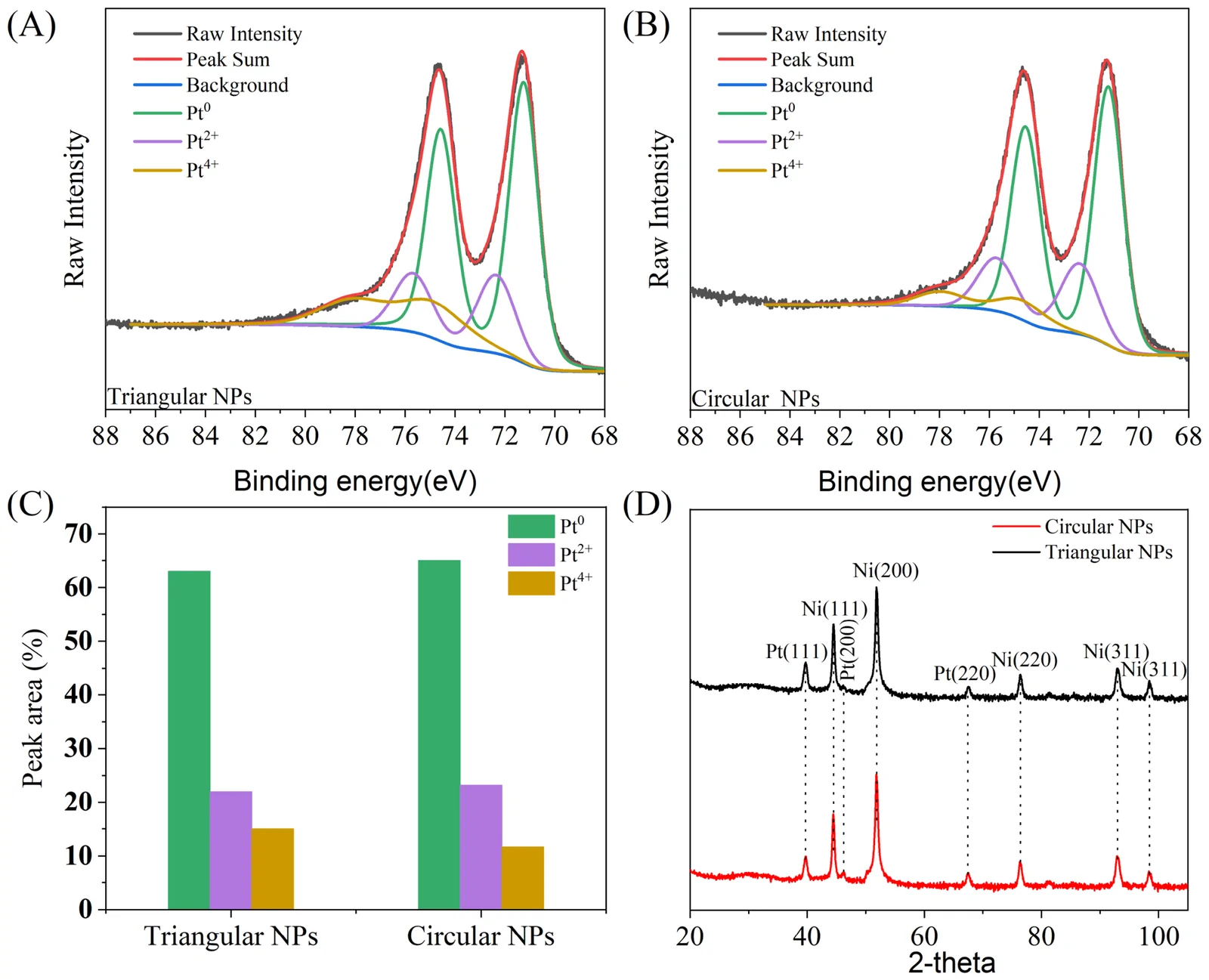
XPS and XRD characterization of triangular and circular Pt/Ni NP arrays. (**A**,**B**) High-resolution Pt 4f XPS spectra and corresponding peak deconvolution of triangular and circular Pt/Ni NPs, respectively. The fitted components are assigned to Pt^0^, Pt^2+^, and Pt^4+^ species. (**C**) Relative peak-area fractions of Pt^0^, Pt^2+^, and Pt^4+^ obtained from the fitted Pt 4f spectra, showing that metallic Pt^0^ is the dominant species in both structures. (**D**) XRD patterns of triangular and circular Pt/Ni NPs, showing similar diffraction peaks assigned to Pt and Ni phases and indicating similar crystalline structures for the two samples.

To further determine whether the different NP geometries introduce changes in the surface chemical state or crystalline structure, XPS and XRD characterizations were performed for both triangular and circular Pt/Ni NPs (Figure 3). The high-resolution Pt 4f spectra of both samples exhibit the characteristic spin-orbit-split Pt 4f_7/2_ and Pt 4f_5/2_ doublets (Figure 3A,B) [27,30]. Each Pt 4f spectrum can be further deconvoluted into three chemical states corresponding to metallic Pt^0^ and oxidized Pt^2+^ and Pt^4+^ species. Oxidized Pt^2+^ and Pt^4+^ species may originate from surface oxidation during air exposure. Metallic Pt^0^ is the dominant component in both samples, indicating that the outer Pt shell is mainly present in the metallic state. Quantitative analysis of the fitted peak areas (Figure 3C) shows that the fractions of Pt^0^, Pt^2+^, and Pt^4+^ are approximately 63%, 22%, and 15% for the triangular NPs and 65%, 23%, and 12% for the circular NPs, respectively [27]. The XPS result indicates that the two samples have similar Pt chemical states.

The crystalline structures of the two Pt/Ni NP arrays were further examined by XRD (Figure 3D). The diffraction peaks at approximately 39.8°, 46.2°, and 67.5° can be assigned to the Pt (111), (200), and (220) planes, respectively [27], while the peaks at approximately 44.5°, 51.8°, 76.4°, 92.9°, and 98.4° correspond to the Ni (111), (200), (220), (311), and (222) planes, respectively [31,32,33]. Importantly, the triangular and circular Pt/Ni NPs show nearly identical diffraction peak positions, with no obvious additional reflections or significant peak shifts. These results indicate that changing the NP cross-sectional geometry does not induce a detectable phase transformation or major lattice variation during fabrication. The crystalline sizes of triangular and circular Pt/Ni NPs were further calculated from the XRD patterns using the Scherrer equation [34], and the values are summarized in Table S1. The calculated crystalline size is 16.56 nm for triangular Pt/Ni NPs and 15.35 nm for circular Pt/Ni NPs. The close crystalline size values demonstrate that the difference in grain size is negligible between the two samples. Together with the XPS results, the XRD analysis confirms that the triangular and circular Pt/Ni NPs possess similar crystalline phases and Pt surface chemical states, providing a reliable basis for evaluating the geometry-dependent electrochemical behavior.

### 3.3. Electrochemical Performance

**Figure 4:**
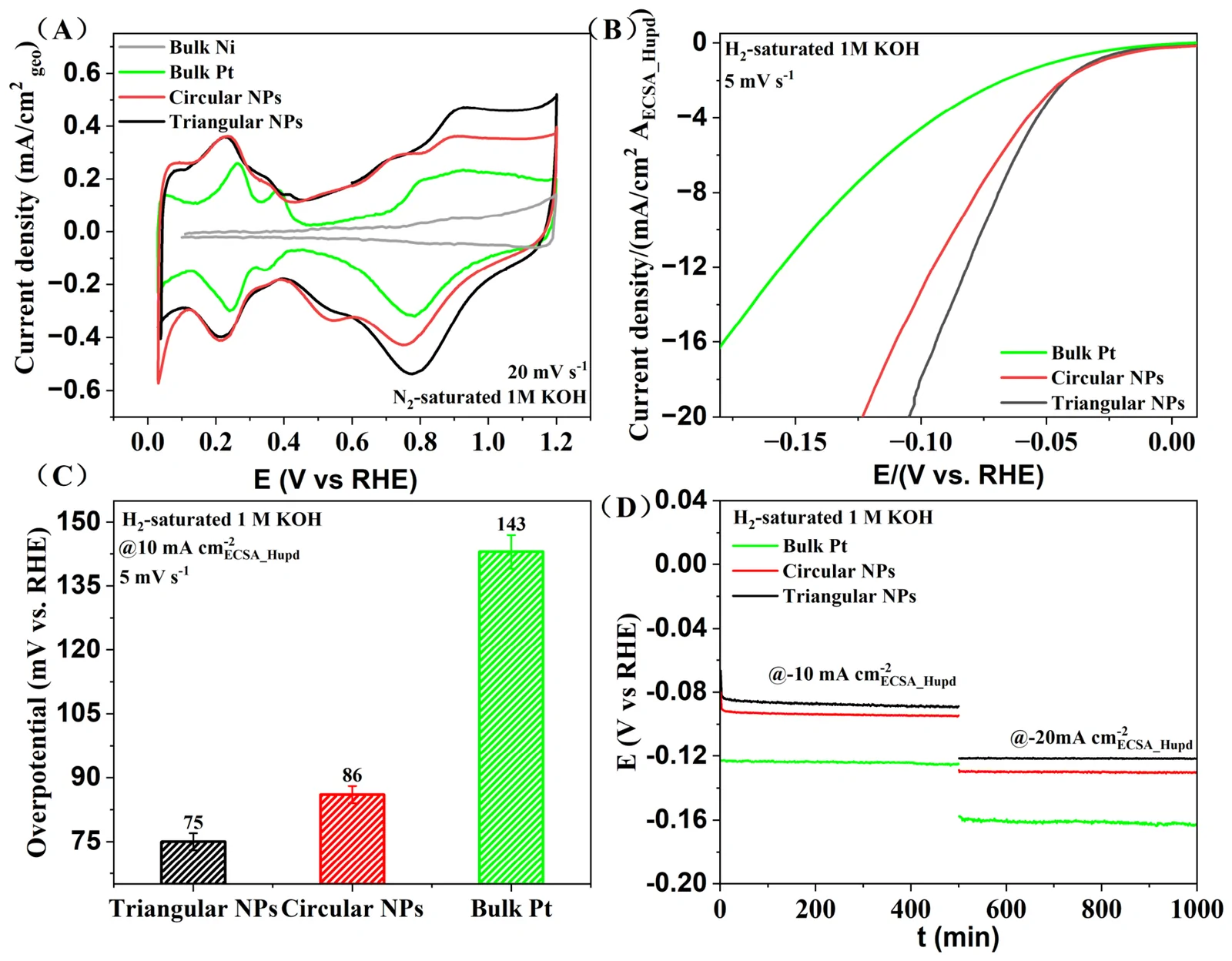
Electrochemical characterization and HER performance of triangular and circular Pt/Ni NP arrays. (**A**) CV curves of triangular Pt/Ni NPs, circular Pt/Ni NPs, bulk Pt, and bulk Ni recorded in N_2_-saturated 1 M KOH at 20 mV s^−1^. (**B**) *ECSA_Hupd_*-normalized HER polarization curves measured in H_2_-saturated 1 M KOH at 5 mV s^−1^ with 85% iR compensation. (**C**) Comparison of overpotentials at 10 mA cm^−2^*_ECSA_*__*Hupd*_. (**D**) Chronopotentiometric stability curves measured at current densities of 10 and 20 mA cm^−2^*_ECSA_*__*Hupd*_ in H_2_-saturated 1 M KOH.

To evaluate the electrocatalytic advantages associated with the triangular geometry, we compared the HER electrocatalytic performance of the triangular and circular Pt/Ni NPs with that of bulk Pt. Figure 4A shows the cyclic voltammetry (CV) curves measured in N_2_-saturated 1 M KOH. Both Pt/Ni NP electrodes exhibit characteristic Pt-related electrochemical features. In the low-potential region, the peaks are mainly associated with hydrogen underpotential deposition (*H_upd_*), corresponding to hydrogen adsorption and desorption on the Pt surface, whereas the peaks at higher potentials are related to the formation and reduction in Pt surface oxides [35,36]. Notably, the triangular and circular Pt/Ni NPs show nearly identical *H_upd_* peak positions and similar Pt-related electrochemical features, suggesting similar Pt surface crystallographic and adsorption environments. This observation is consistent with the nearly identical Pt diffraction peak positions observed by XRD. Importantly, bulk Ni exhibits only a weak and featureless response in the Pt *H_upd_* potential region, with no redox peaks overlapping the *H_upd_* features of the Pt-containing electrodes, indicating that Ni redox processes make no obvious overlapping contribution to the integrated *H_upd_* charge under these conditions. This supports the use of *H_upd_* as a comparative *ECSA* metric for the Pt-based electrodes, yielding comparable *ECSA* values of 1.31 ± 0.04, 1.40 ± 0.05 and 1.25 ± 0.03 cm^2^ for the triangular and circular NPs and bulk Pt, respectively.

The HER activity was further evaluated by linear sweep voltammetry (LSV). The measurements were performed in H_2_-saturated 1 M KOH using a reversible hydrogen electrode (RHE) as the reference electrode [27]. The current densities were normalized by the *ECSA* determined from the *H_upd_* region to account for differences in electrochemically accessible Pt surface area and thereby enable a more meaningful comparison of the geometry-dependent HER activity. As shown in Figure 4B, the triangular Pt/Ni NPs exhibit the most positive polarization curve and require the lowest overpotential. At 10 mA cm^−2^*_ECSA_*__*Hupd*_, the overpotentials are 75 ± 2, 86 ± 2, and 143 ± 4 mV for the triangular Pt/Ni NPs, circular Pt/Ni NPs, and bulk Pt, respectively (Figure 4C). The corresponding Tafel plots are shown in Figure S15. The triangular Pt/Ni NPs exhibit the lowest Tafel slope of 40 mV dec^−1^, compared with 45.1 mV dec^−1^ for the circular NPs and 60.8 mV dec^−1^ for bulk Pt, indicating faster HER kinetics for the triangular structure.

To facilitate comparison with conventional HER electrocatalysts, the polarization curves were also normalized to the geometric electrode area (Figure S13). At 10 mA cm^−2^_geo_, the triangular and circular Pt/Ni NPs exhibit overpotentials of 59 and 65 mV, respectively. A comparison with representative Pt-based HER catalysts reported in alkaline electrolytes is summarized in Table S2. The geometric-area-normalized performance of the triangular NPs is comparable to that of several reported Pt-based catalysts.

The electrochemical stability of the electrodes was further evaluated by chronopotentiometry at constant *ECSA*-normalized current densities (Figure 4D). The electrodes were first operated at 10 mA cm^−2^*_ECSA_*__*Hupd*_ for 500 min and subsequently at 20 mA cm^−2^*_ECSA_*__*Hupd*_ for an additional 500 min. All three electrodes show relatively stable potentials during both current-density stages, indicating good HER stability. Notably, the triangular Pt/Ni NPs retain their enhanced activity throughout the prolonged electrolysis. To directly probe potential drift and surface electrochemical changes induced by long-term electrolysis, CV and LSV measurements were performed before and after the 1000-min stability test for triangular Pt/Ni NPs (Figure S16). The pre- and post-stability CV curves acquired in N_2_-saturated 1 M KOH exhibit highly overlapping redox features, and the corresponding HER LSV polarization curves display negligible potential shift, confirming minimal potential drift and well-maintained electrochemical properties after prolonged operation. Post-stability SEM characterization further shows that the ordered NP arrangement and triangular morphology are largely preserved after the electrochemical test, with no obvious structural collapse or severe aggregation observed (Figure S17). These results support electrochemical durability and morphological retention under the tested HER conditions.

Taken together, the triangular Pt/Ni NPs exhibit higher HER activity and more favorable reaction kinetics than their circular counterparts, despite their comparable Pt chemical states, diffraction features, local Pt (111) d-spacings, and *ECSA* values. Although the Ni core may contribute to alkaline HER, particularly by facilitating water dissociation, the comparable Pt/Ni core-shell architectures and identical Ni electrodeposition conditions suggest that its contribution should be similar for both geometries and is therefore unlikely to account for the observed geometry-dependent enhancement. Moreover, the comparable *ECSA* values indicate that the enhanced *ECSA*-normalized HER performance cannot be simply attributed to a substantially larger electrochemically accessible Pt surface area. Instead, we attribute the enhanced HER performance primarily to the sharp nanoedges, which modulate the local electrode/electrolyte interfacial environment. To further clarify the physical origin of such HER enhancement, COMSOL simulations were subsequently employed to compare the electric field and current-density distributions around the triangular and circular Pt/Ni NPs.

### 3.4. COMSOL Simulation

To gain physical insight into the geometry-dependent interfacial environment, steady-state two-dimensional COMSOL Multiphysics simulations were carried out using the tertiary-current-distribution (Nernst-Planck) interface, which couples electrolyte mass transport and Butler–Volmer HER kinetics at −50 mV vs. RHE, closely matching experimental operating conditions. Identical electrolyte properties, kinetic parameters and boundary conditions were set for triangular and circular computational domains; only the geometry was varied, mirroring our experimental control design.

**Figure 5:**
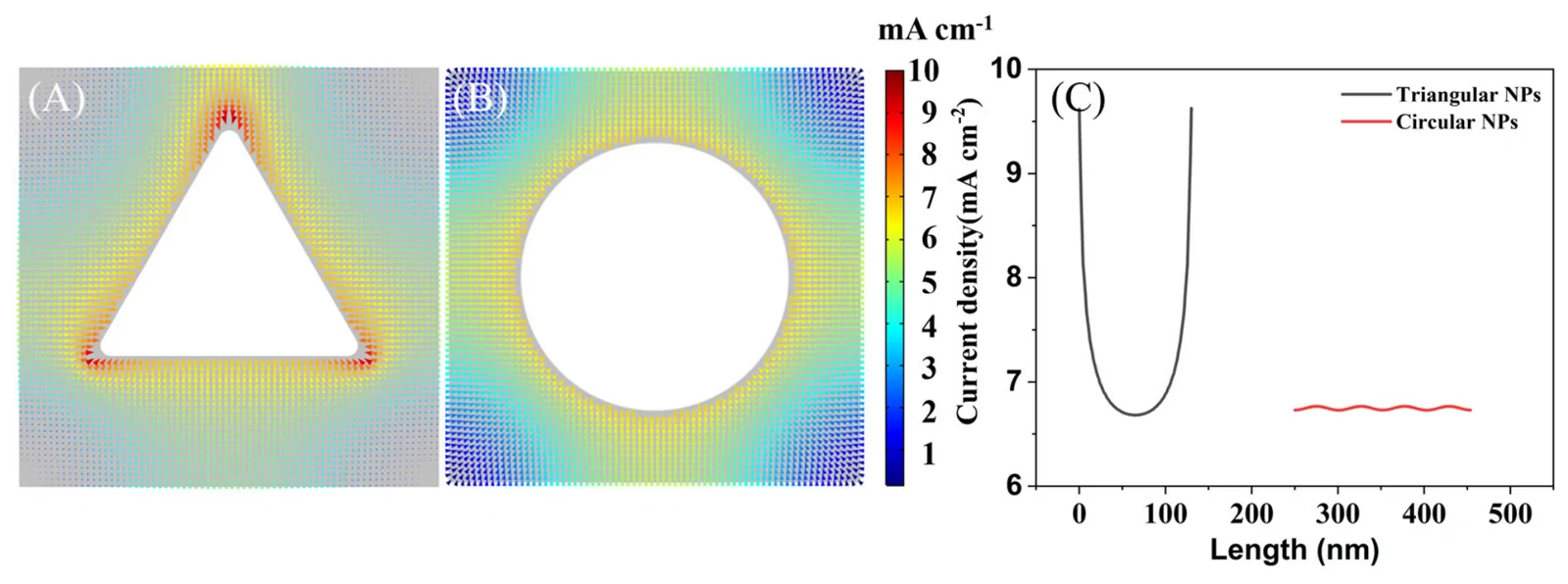
COMSOL simulation of the geometry-dependent current-density distribution around triangular and circular Pt/Ni NPs. (**A**,**B**) Simulated current-density distributions around triangular and circular Pt/Ni NPs, respectively. Pronounced current-density concentration is observed at the high-curvature corners of the triangular NP, whereas a more uniform current distribution is obtained around the circular NP. (**C**) Corresponding current-density profiles along the nanopillar/electrolyte interface, showing substantially enhanced local current density at the corners of the triangular NP compared with the circular counterpart. The geometry-induced current concentration provides mechanistic support for the experimentally observed geometry-dependent HER enhancement.

The influence of nanopillar geometry on the local electric-field distribution was first investigated (Figure S18). The circular nanopillar exhibits a relatively uniform electric-field distribution, whereas pronounced electric-field concentration occurs at the sharp corners of the triangular nanopillar due to the high curvature of the nanoedges. Such geometry-induced field localization may modify the local interfacial electrochemical environment, including ionic transport and charge-transfer behavior, thereby contributing to the enhanced catalytic response of the triangular nanopillars [7,8]. Furthermore, the local current-density distributions were simulated to directly evaluate the influence of geometry on electrochemical reaction activity (Figure 5). Compared with the circular nanopillar, the triangular nanopillar exhibits significant current-density concentration near the sharp corners. The maximum local interfacial current density at the triangular corners is approximately 41% higher than that around the circular nanopillar surface. Since the triangular and circular Pt/Ni NPs have similar crystalline phases and Pt chemical states, the difference in HER performance is unlikely to arise primarily from major material differences. Instead, the improved catalytic activity can be attributed primarily to the geometry-induced modification of the local electrochemical environment, where the sharp nanoedges promote electric-field and current-density localization during HER operation.

## 4. Conclusions

In this work, vertically ordered triangular Pt/Ni NP arrays with sharp nanoedges were fabricated by an AAO template-assisted strategy, and comparison with matched circular Pt/Ni NP arrays demonstrated that the nanoedge geometry itself enhances alkaline HER activity by 35% at −100 mV vs. RHE. This overall result is based on the following findings:

(1) Triangular Pt-shell/Ni-core NP arrays (side length: ~130 nm, corner radius: ~5 nm) with ordered domains of up to 1 cm were obtained by replicating triangular AAO nanopores, together with circular control arrays (diameter: ~140 nm) prepared under identical conditions.
(2) The two arrays differ mainly in geometry, showing comparable Pt loadings (63.4 vs. 63.9 μg cm^−2^), crystalline phases, Pt chemical states, and *ECSA* values (1.31 vs. 1.40 cm^2^).
(3) The triangular NPs require an *ECSA*-normalized overpotential of 75 mV at 10 mA cm^−2^, compared with 86 mV for circular NPs and 143 mV for bulk Pt, with the lowest Tafel slope (40 mV dec^−1^) and stable operation for 1000 min.
(4) COMSOL simulations reveal electric-field and current-density localization at the sharp corners, with a maximum local current density ~41% higher than that of the circular NP, explaining the enhanced activity.

These results identify nanoscale geometry as a complementary design parameter for electrocatalysts beyond compositional optimization.

## Data Availability

The raw data supporting the conclusions of this article will be made available by the authors on request.

## Supplementary Materials

The following supporting information can be downloaded at: https://www.mdpi.com/article/10.3390/nano16211395/s1, Figure S1. (A) Schematic illustration and (B) photograph of the custom-built electrochemical anodization cell for fabricating AAO templates. The pre-patterned Al foil acts as the anode and is in good thermal and electrical contact with a copper cooling plate with circulating cooling water for precise temperature control during anodization. A Pt wire is used as the cathode, and the cell is driven by a constant-voltage DC power supply; Figure S2. Cross-sectional SEM image of the AAO template with triangular nanopores; Figure S3. Statistical distribution of side length for the triangular AAO templates; Figure S4. Low-magnification SEM image demonstrating the large-area uniformity and periodicity of the triangular Pt/Ni nanopillar arrays over a lateral distance of approximately 24 μm; Figure S5. Top-view STEM and corresponding EDX elemental mapping of triangular Pt/Ni core-shell nanopillar arrays. (A) Top-view STEM image of the periodic triangular NP array. (B) Overlaid EDX elemental map of Pt and Ni. (C) Ni elemental distribution map. (D) Pt elemental distribution map. The Pt shell fully covers the nanopillar tips without observable exposed Ni, confirming complete encapsulation of the Ni core; Figure S6. (A) Cross-sectional STEM images and (B) corresponding EDX line-scan profiles of triangular Pt/Ni NPs; Figure S7. SEM and pore-size distribution of the AAO template with circular nanopores. (A) Top-view SEM image showing the highly ordered arrangement and uniform morphology of the circular nanopores. (B) Statistical distribution of the pore diameter, yielding an average value of approximately 140 nm; Figure S8. SEM characterization of circular Pt/Ni nanopillar arrays. (A) Top-view and (B) tilted-view SEM images of the circular Pt/Ni nanopillar arrays, showing their highly ordered arrangement and good structural uniformity; Figure S9. Statistical distribution of the circular nanopillar diameter, showing an average value of approximately 140 nm; Figure S10. (A) Cross-sectional STEM images and (B) corresponding EDX line-scan profiles of circular Pt/Ni NPs; Figure S11. Structural and compositional characterization of circular Pt/Ni nanopillars. (A) STEM image of circular Pt/Ni nanopillars, with the yellow dashed box indicating the region selected for high-resolution analysis. (B) Overlaid EDX elemental map of Ni and Pt. (C, D) Individual EDX maps of Ni and Pt, respectively, showing a Pt-shell/Ni-core elemental distribution. (E) HRTEM image acquired from the selected region in (A), exhibiting lattice fringes with an interplanar spacing of approximately 0.226 nm, consistent with the Pt (111) planes. (F) Corresponding FFT pattern confirming the crystalline nature of the Pt-containing shell region; Figure S12. Cell voltage–time curve recorded during Ni electrodeposition at a constant current density of −2 mA cm^−2^ for 2400 s; Figure S13. Geometric current-density-normalized HER polarization curves of triangular and circular Pt/Ni NP arrays. The measurements were performed in H_2_-saturated 1 M KOH at a scan rate of 5 mV s^−1^ with 85% iR compensation; Figure S14. *Hupd* integration and baseline subtraction for *ECSA* determination. Representative CV curves of (A) triangular and (B) circular Pt/Ni NPs recorded in N2-saturated 1 M KOH at a scan rate of 20 mV s^−1^ are shown. The *Hupd* region and the corresponding baseline used for background-current subtraction are indicated; Figure S15. Tafel plots of triangular Pt/Ni nanopillars, circular Pt/Ni nanopillars, and bulk Pt for the hydrogen evolution reaction. The triangular and circular Pt/Ni nanopillars and bulk Pt exhibit Tafel slopes of 40, 45.1 and 60.8 mV dec^−1^, respectively. The lower Tafel slope of the triangular Pt/Ni nanopillars indicates faster HER kinetics compared with the circular counterpart; Figure S16. (A) Cyclic voltammetry (CV) curves of triangular NPs recorded in N_2_-saturated 1 M KOH at 20 mV s^−1^ before and after the 1000-min stability test. (B) Corresponding LSV polarization curves in H_2_-saturated 1 M KOH at 5 mV s^−1^ before and after stability testing. The nearly overlapping CV and LSV profiles demonstrate negligible potential drift and good electrocatalytic stability of the triangular NPs after long-term operation; Figure S17. SEM characterization of the triangular Pt/Ni nanopillar arrays after the electrochemical stability test, showing the ordered arrangement and sharp nanoedge morphology remaining intact and no obvious structural degradation observed; Figure S18. COMSOL-simulated electric-field distributions around triangular and circular Pt/Ni nanopillars, showing pronounced electric-field enhancement at the sharp nanocorners of the triangular nanopillars; Table S1. Summary of electrochemical and structural parameters of triangular and circular Pt/Ni NPs; Table S2. Comparison of HER performance of triangular and circular Pt/Ni NPs with representative Pt-based HER electrocatalysts reported in alkaline electrolytes; Table S3. Parameters used in the COMSOL Multiphysics simulation.
