# Supplementary material for: AAO Template-Assisted Fabrication of Vertically Ordered Triangular Pt/Ni Nanopillar Arrays for Enhanced Hydrogen Evolution Reaction
Source: Nanomaterials (Basel). 2026 Oct 30;16(21):1395. doi: 10.3390/nano16211395 (PMC13765106; doi:10.3390/nano16211395)
Supplement: Supplementary file 1 [file nanomaterials-16-01395-s001.zip › nanomaterials-4592742-supplementary.pdf]

AAO Template-Assisted Fabrication of Vertically Ordered Triangular Pt/Ni Nanopillar Arrays for Enhanced Hydrogen Evolution Reaction

Zhiqiang Zeng[1,†*], Mingshu Zheng[2,†], Lin Yang[3*],Rui Xu[4*]

[1]National Laboratory of Solid State Microstructures, School of Physics, Collaborative Innovation Center of Advanced Microstructures, Jiangsu Physical Science Research Center, Nanjing University, Nanjing, 210093, China.

[2]School of Food Science and Pharmaceutical Engineering, Nanjing Normal University, 210023, China.

[3]Academician Expert Workstation, Jinhua University of Vocational Technology, Jinhua, 321017, Zhejiang, China

[4]Helmholtz-Zentrum Dresden-Rossendorf e.V. (HZDR), Institute of Ion Beam Physics and Materials Research, Germany

[†]These authors contributed equally to this work.

[*]Corresponding authors: zhiqiang.zeng@nju.edu.cn, 20231039@jhc.edu.cn, r.xu@hzdr.de.

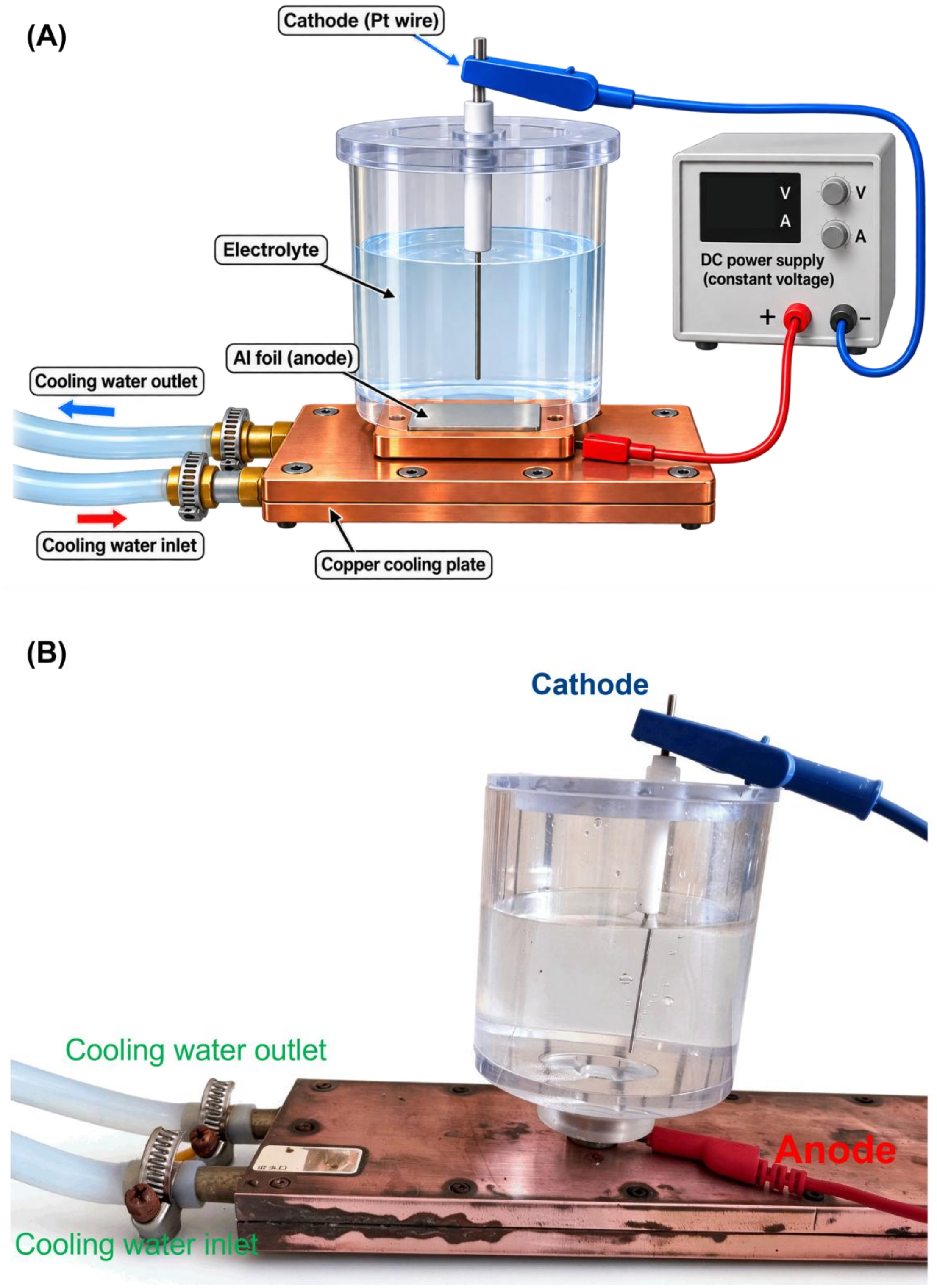

Figure S1. (A) Schematic illustration and (B) photograph of the custom-built electrochemical anodization cell for fabricating AAO templates. The pre-patterned Al foil acts as the anode and is in good thermal and electrical contact with a copper cooling plate with circulating cooling water for precise temperature control during anodization.

A Pt wire is used as the cathode, and the cell is driven by a constant-voltage DC power supply.

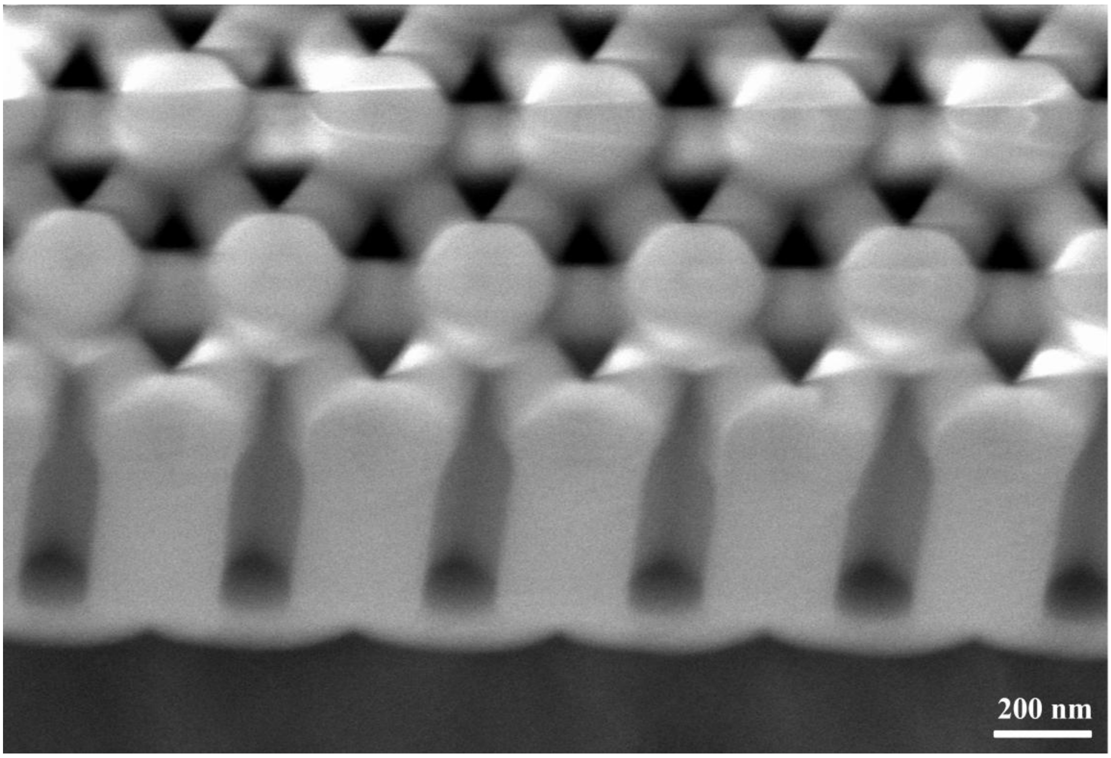

Figure S2. Cross-sectional SEM image of the AAO template with triangular nanopores.

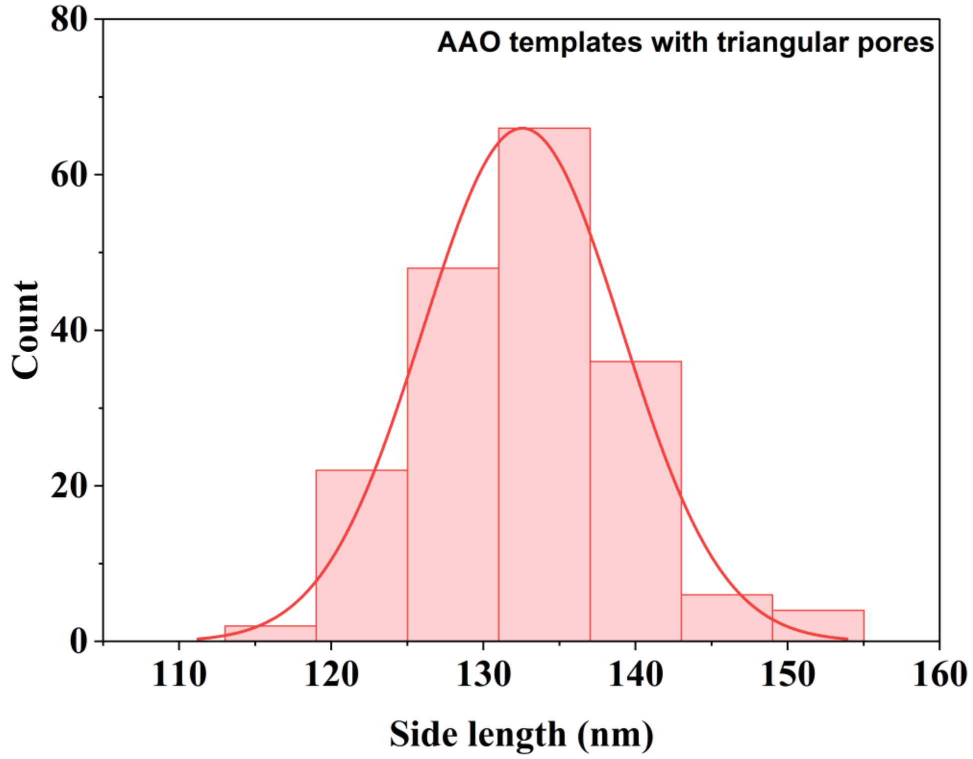

Figure S3. Statistical distribution of side length for the triangular AAO templates.

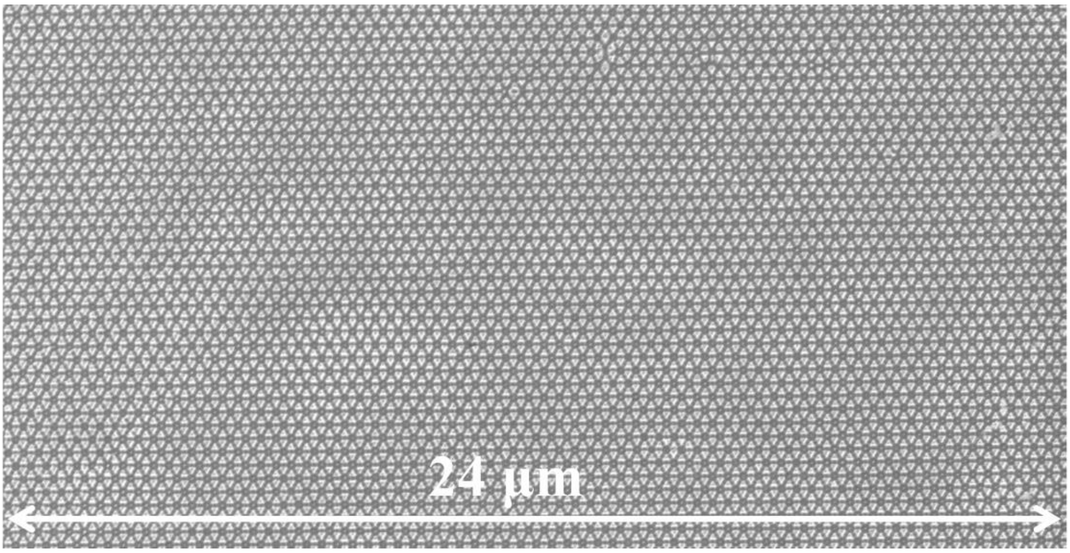

Figure S4. Low-magnification SEM image demonstrating the large-area uniformity and periodicity of the triangular Pt/Ni nanopillar arrays over a lateral distance of approximately 24 μm.

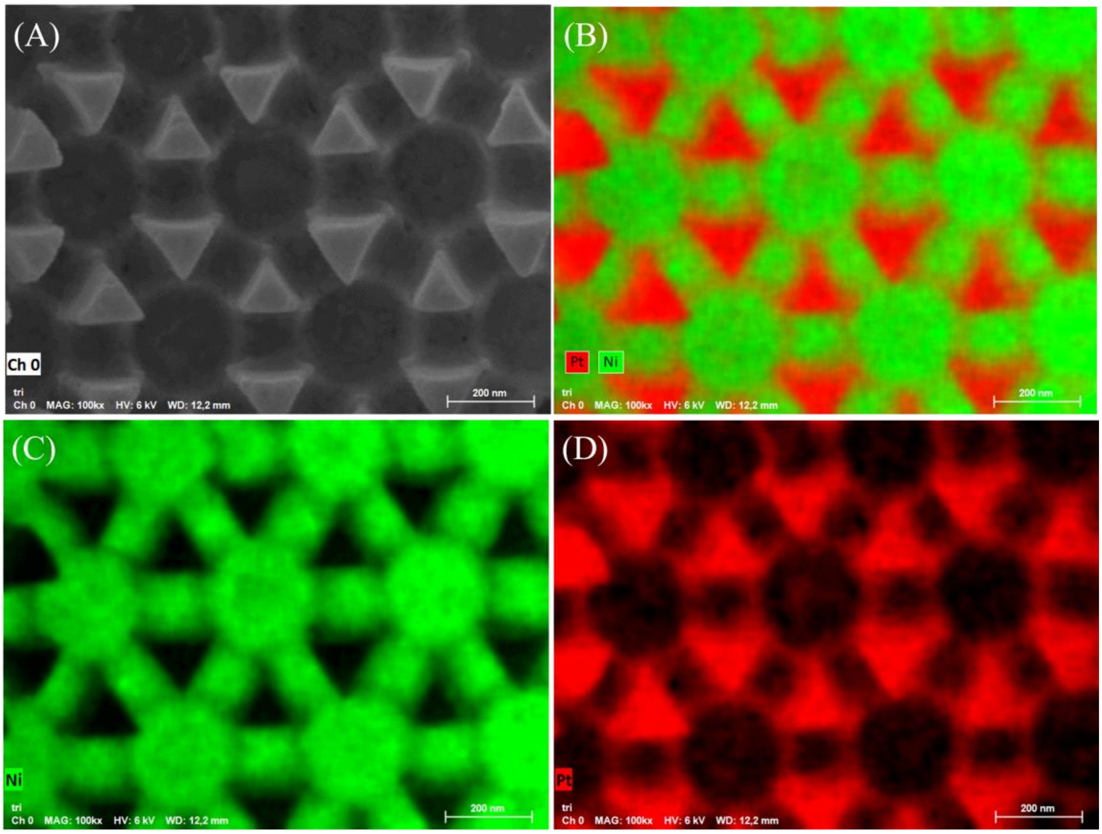

Figure S5. Top-view STEM and corresponding EDX elemental mapping of triangular Pt/Ni core-shell nanopillar arrays. (A) Top-view STEM image of the periodic triangular NP array. (B) Overlaid EDX elemental map of Pt and Ni. (C) Ni elemental distribution map. (D) Pt elemental distribution map. The Pt shell fully covers the nanopillar tips without observable exposed Ni, confirming complete encapsulation of the Ni core.

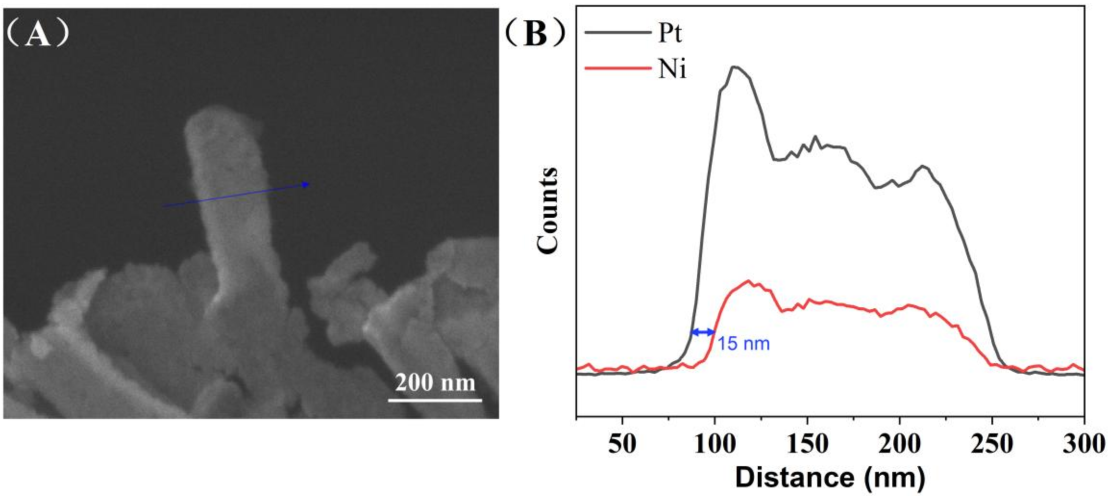

Figure S6. (A) Cross-sectional STEM images and (B) corresponding EDX line-scan profiles of triangular Pt/Ni NPs.

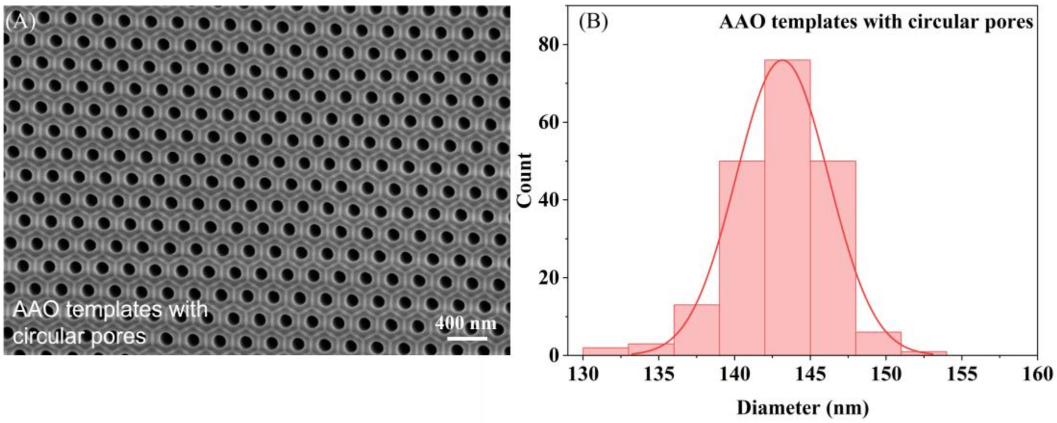

Figure S7. SEM and pore-size distribution of the AAO template with circular nanopores. (A) Top-view SEM image showing the highly ordered arrangement and uniform morphology of the circular nanopores. (B) Statistical distribution of the pore diameter, yielding an average value of approximately 140 nm.

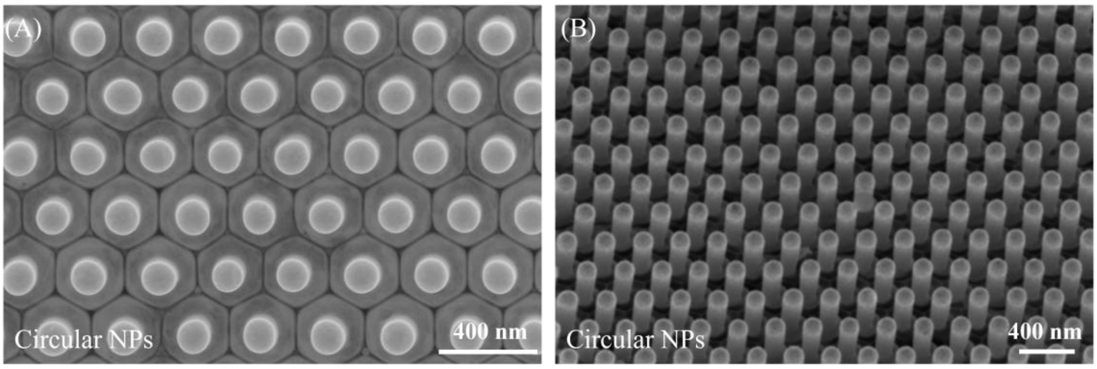

Figure S8. SEM characterization of circular Pt/Ni nanopillar arrays. (A) Top-view and (B) tilted-view SEM images of the circular Pt/Ni nanopillar arrays, showing their highly ordered arrangement and good structural uniformity.

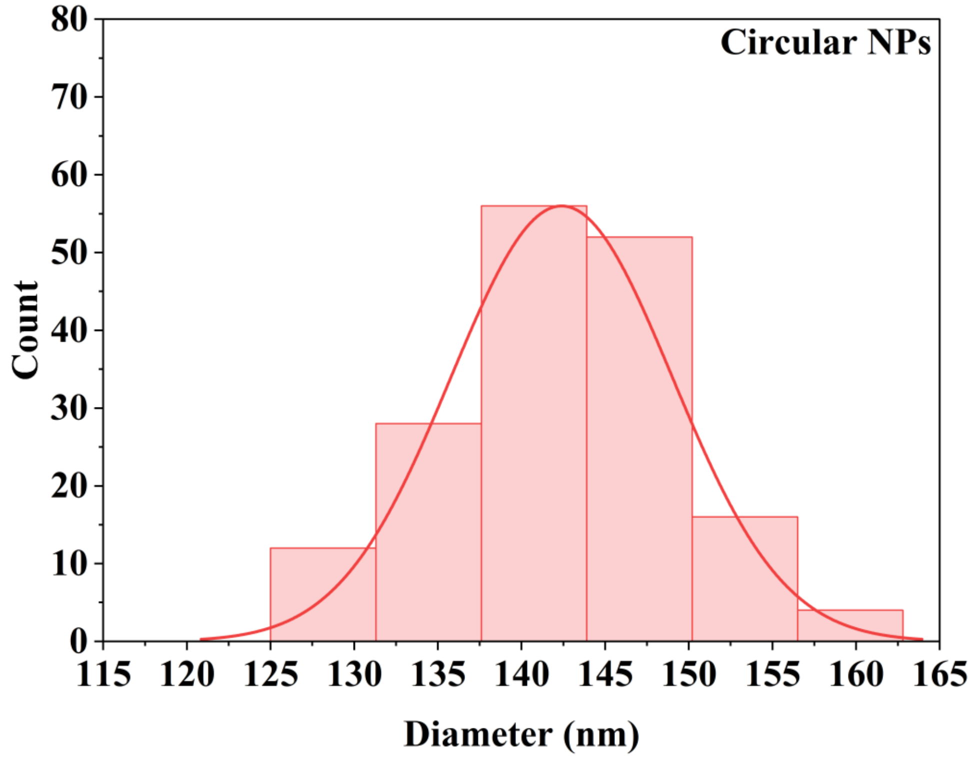

Figure S9. Statistical distribution of the circular nanopillar diameter, showing an average value of approximately 140 nm.

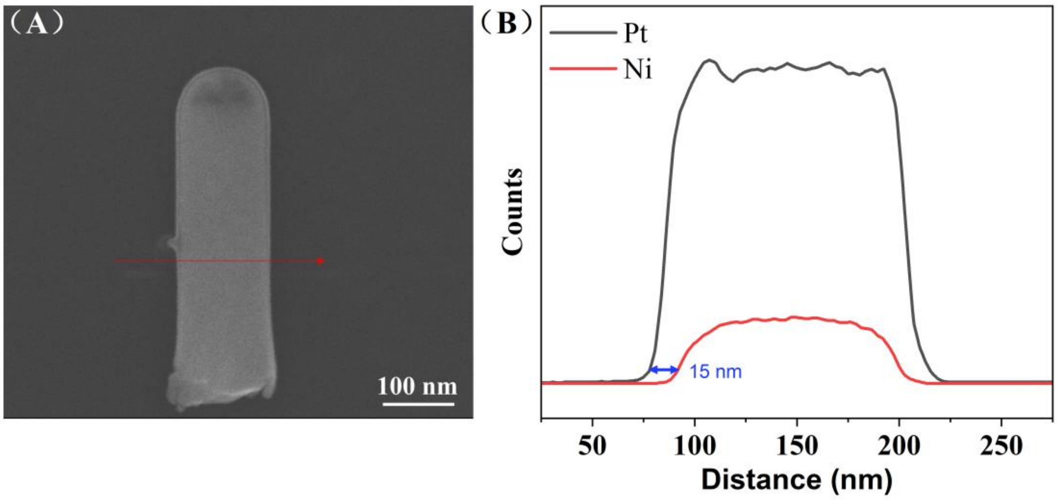

Figure S10. (A) Cross-sectional STEM images and (B) corresponding EDX line-scan profiles of circular Pt/Ni NPs.

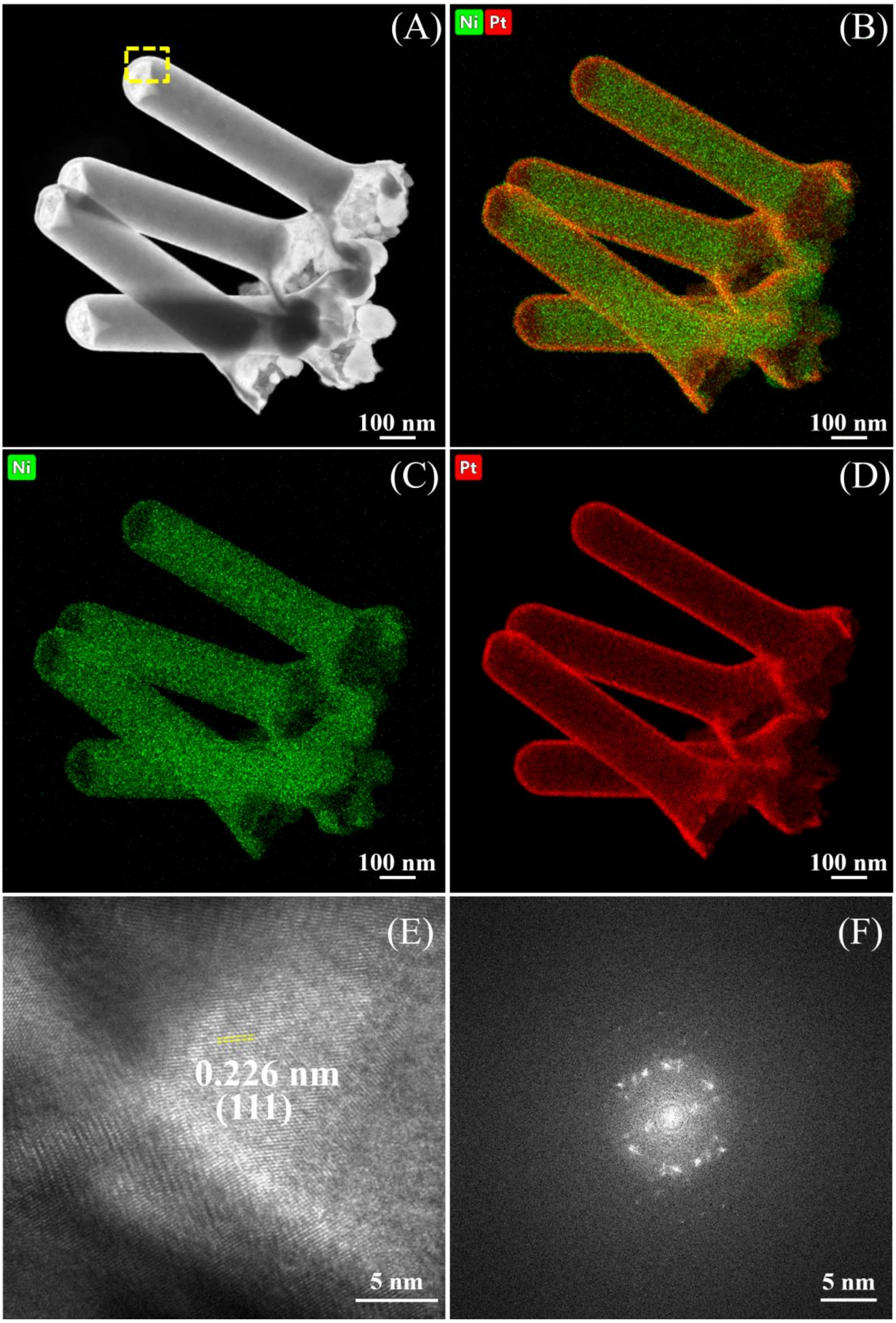

Figure S11. Structural and compositional characterization of circular Pt/Ni nanopillars. (A) STEM image of circular Pt/Ni nanopillars, with the yellow dashed box indicating the region selected for high-resolution analysis. (B) Overlaid EDX elemental map of Ni and Pt. (C, D) Individual EDX maps of Ni and Pt, respectively, showing a Pt-shell/Ni-core elemental distribution. (E) HRTEM image acquired from the selected

region in (A), exhibiting lattice fringes with an interplanar spacing of approximately 0.226 nm, consistent with the Pt (111) planes. (F) Corresponding FFT pattern confirming the crystalline nature of the Pt-containing shell region.

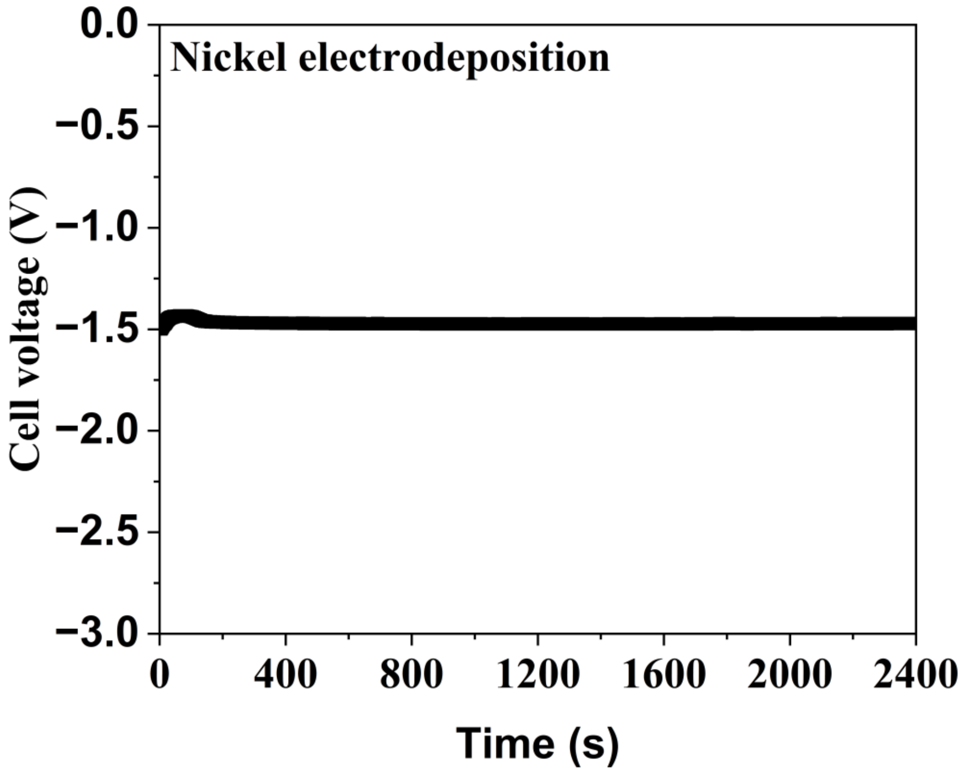

Figure S12. Cell voltage–time curve recorded during Ni electrodeposition at a constant current density of −2 mA cm^{-2} for 2400 s.

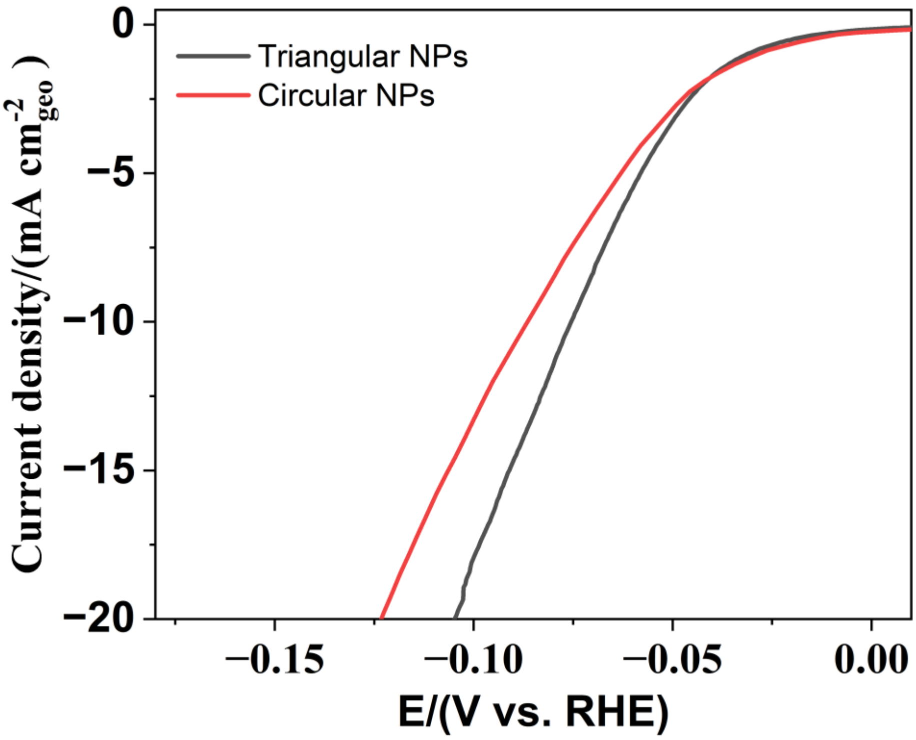

Figure S13. Geometric current-density-normalized HER polarization curves of triangular and circular Pt/Ni NP arrays. The measurements were performed in H_2-saturated 1 M KOH at a scan rate of 5 mV s^{-1} with 85% iR compensation.

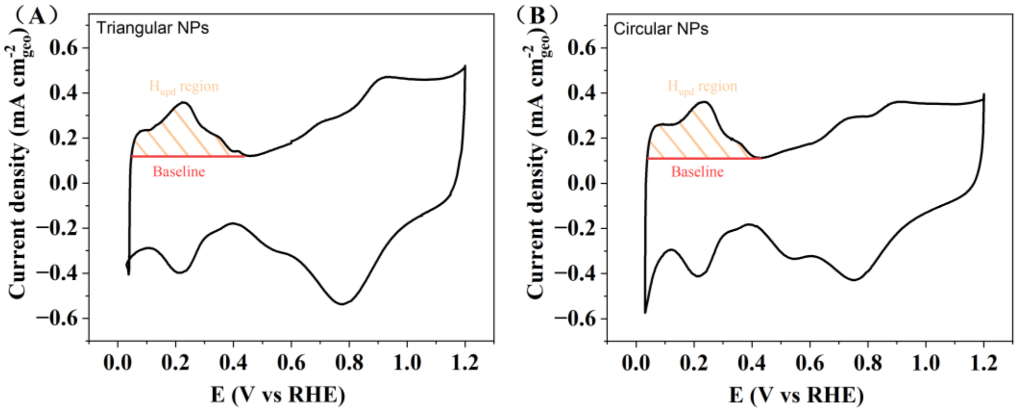

Figure S14. H_{upd} integration and baseline subtraction for ECSA determination. Representative CV curves of (A) triangular and (B) circular Pt/Ni NPs recorded in N_2-saturated 1 M KOH at a scan rate of 20 mV s^{-1} are shown. The H_{upd} region and the corresponding baseline used for background-current subtraction are indicated.

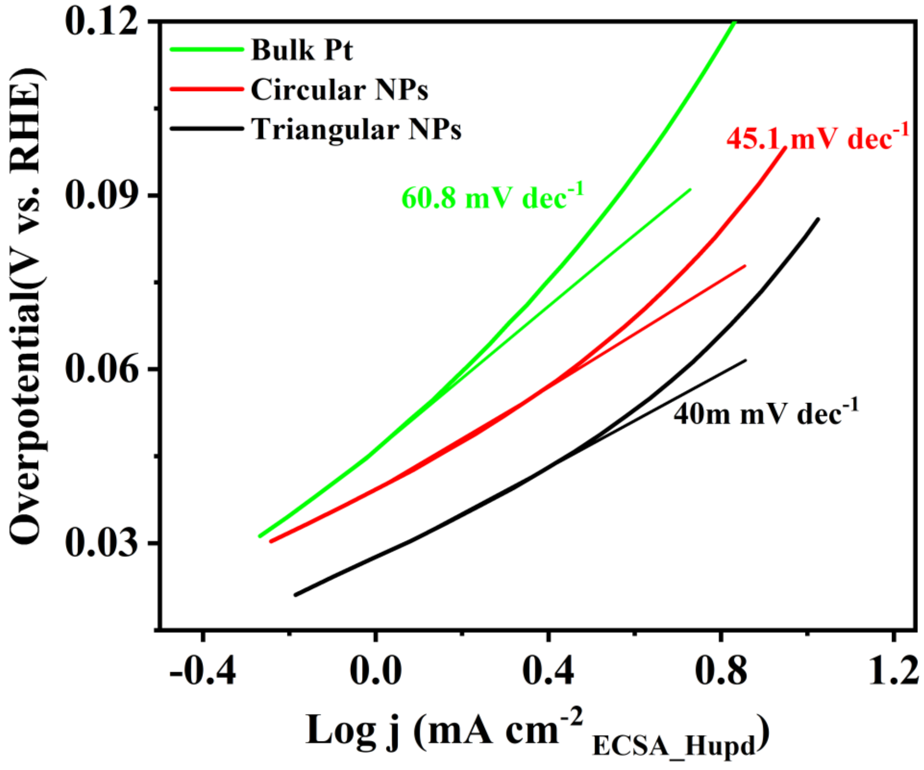

Figure S15. Tafel plots of triangular Pt/Ni nanopillars, circular Pt/Ni nanopillars, and bulk Pt for the hydrogen evolution reaction. The triangular and circular Pt/Ni nanopillars and bulk Pt exhibit Tafel slopes of 40, 45.1 and 60.8 mV dec^{-1}, respectively. The lower Tafel slope of the triangular Pt/Ni nanopillars indicates faster HER kinetics compared with the circular counterpart.

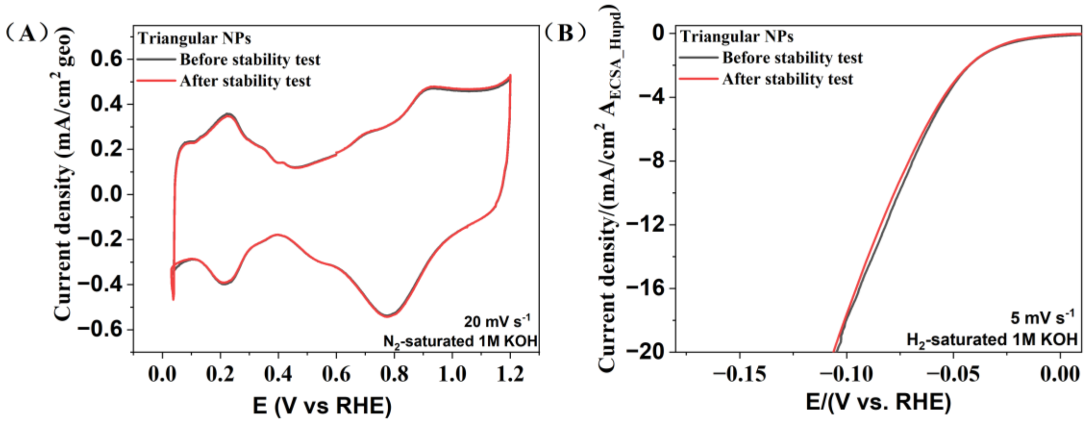

Figure S16. (A) Cyclic voltammetry (CV) curves of triangular NPs recorded in N_2-saturated 1 M KOH at 20 mV s^{-1} before and after the 1000-min stability test. (B) Corresponding LSV polarization curves in H_2-saturated 1 M KOH at 5 mV s^{-1} before and after stability testing. The nearly overlapping CV and LSV profiles demonstrate negligible potential drift and good electrocatalytic stability of the triangular NPs after long-term operation.

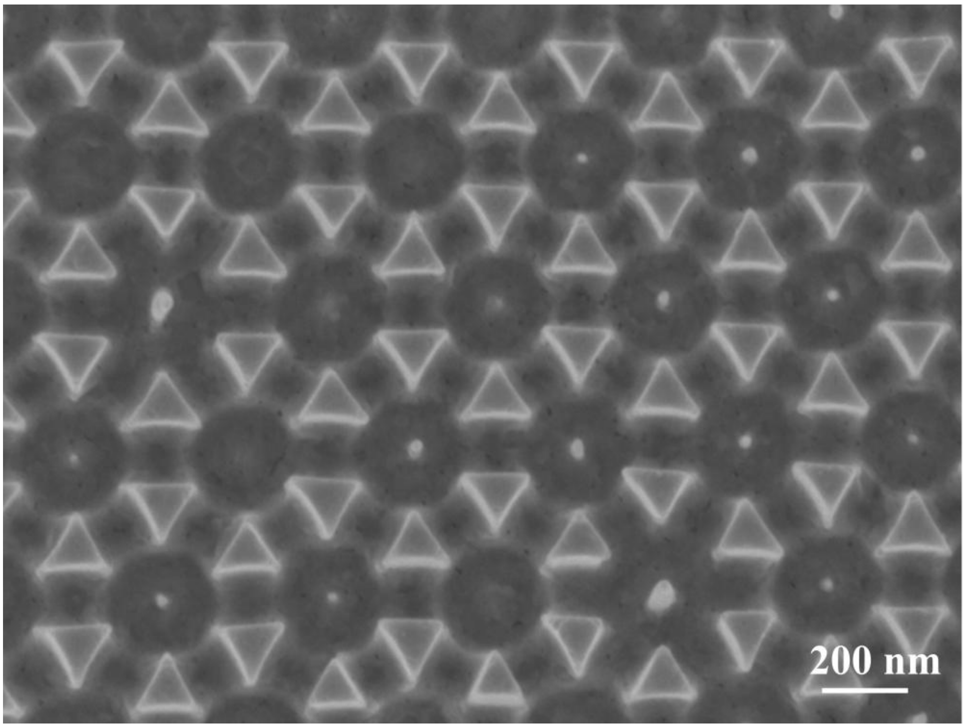

Figure S17. SEM characterization of the triangular Pt/Ni nanopillar arrays after the electrochemical stability test, showing the ordered arrangement and sharp nanoedge morphology remaining intact and no obvious structural degradation observed.

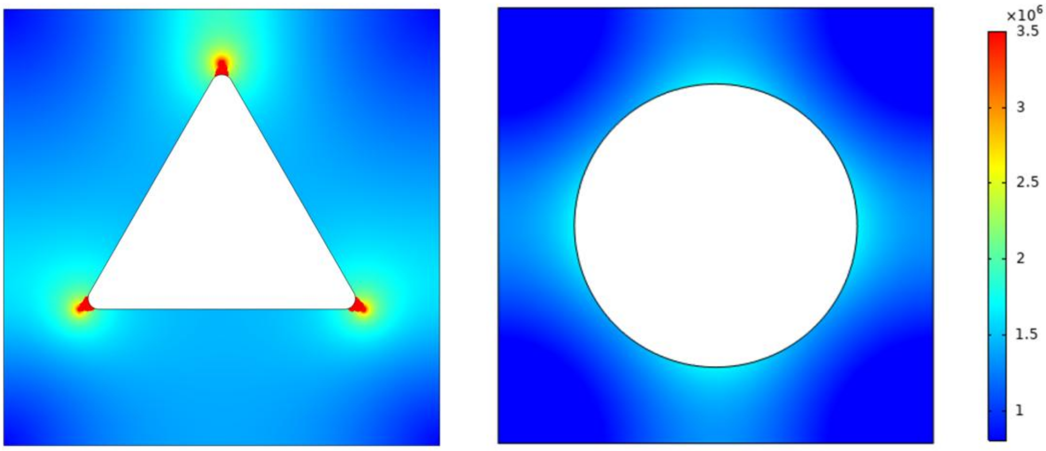

Figure S18. COMSOL-simulated electric-field distributions around triangular and circular Pt/Ni nanopillars, showing pronounced electric-field enhancement at the sharp nanocorners of the triangular nanopillars.

Table S1. Summary of electrochemical and structural parameters of triangular and circular Pt/Ni NPs.

Sample	Triangular NPs	Circular NPs
[a]ECSA_#1 (cm^2)	1.28	1.34
[a]ECSA_#2 (cm^2)	1.30	1.41
[a]ECSA_#3 (cm^2)	1.35	1.45
Average ECSA (cm^2)	1.31 ± 0.04	1.40 ± 0.05
[b]Roughness factor	1.9 ± 0.05	2.0 ± 0.07
[c]Crystalline size (nm)	16.56	15.35
Pt mass loading_#1 ($\mu g\ cm^{-2}$)	62.8	63.1
Pt mass loading_#2 ($\mu g\ cm^{-2}$)	63.4	64.1
Pt mass loading_#3 ($\mu g\ cm^{-2}$)	63.9	64.5
Average Pt mass loading ($\mu g\ cm^{-2}$)	63.4 ± 0.6	63.9 ± 0.7

a: Roughness factor and ECSA are based on the H desorption peak.
b: Roughness factor=ECSA/A_{geo}
c: Crystalline size was calculated by the Scherrer equation from XRD data.

Table S2. Comparison of HER performance of triangular and circular Pt/Ni NPs with representative Pt-based HER electrocatalysts reported in alkaline electrolytes.

Catalyst	η_{10_ECSA} (mV)	η_{10_geo} (mV)	Electrolyte	Reference
Triangular NPs	75	59	1.0 M KOH	This work
Circular NPs	86	65	1.0 M KOH	This work
Pt/LiCoO2	NA	61	1.0 M KOH	[1]
Pt/N-Mo2C	NA	100	1.0 M KOH	[2]
10-Pt/Ni–P	NA	87	1.0 M KOH	[3]
Pt/Co_2P/Ni_2P/NF	NA	75	1.0 M KOH	[4]
Pt/NWs/SL-Ni(OH)2	120	85.5	1.0 M KOH	[5]
hcp Pt–Ni	65	~90	0.1 M KOH	[6]

Table S3. Parameters used in the COMSOL Multiphysics simulation.

Name	Value
Side length of triangles	1.30×10^{-7} m
Corner curvature radius	5×10^{-9} m
Diameter of circles	1.40×10^{-7} m
Temperature	298.15 K
Electrode potential	-50 mV
Diffusion coefficient of OH^-	5.30×10^{-9} m^2 s^{-1}
Diffusion coefficient of K^+	3.75×10^{-9} m^2 s^{-1}
Bulk OH^- concentration	1000 mol m^{-3}
Bulk K^+ concentration	1000 mol m^{-3}
K^+ ion charge number	1
OH^- ion charge number	-1
Bulk ionic strength	1000 mol m^{-3}
Relative permittivity of water	78.5
Debye length	3.0421×10^{-10} m
Stern layer thickness	5.00×10^{-10} m

Reference

1. Zheng, X.; Cui, P.; Qian, Y.; Zhao, G.; Zheng, X.; Xu, X.; Cheng, Z.; Liu, Y.; Dou, S.X.; Sun, W. Multifunctional Active-Center-Transferable Platinum/Lithium Cobalt Oxide Heterostructured Electrocatalysts towards Superior Water Splitting. *Angew. Chem.* **2020**, *132*, 14641-14648.
2. Qiu, Y.; Wen, Z.; Jiang, C.; Wu, X.; Si, R.; Bao, J.; Zhang, Q.; Gu, L.; Tang, J.; Guo, X. Rational Design of Atomic Layers of Pt Anchored on Mo2C Nanorods for Efficient Hydrogen Evolution over a Wide pH Range. *Small* **2019**, *15*, 1900014.
3. Battiato, S.; Bruno, L.; Terrasi, A.; Mirabella, S. Superior Performances of Electroless-Deposited Ni–P Films Decorated with an Ultralow Content of Pt for Water-Splitting Reactions. *ACS Applied Energy Materials* **2022**, *5*, 2391-2399.
4. Xiao, X.; Sun, D.; Liu, X.; Qiu, B.; Xu, X.; Zhang, D.; Yang, T. Ultra-low amount Pt-doped Co2P/Ni2P on nickel foam as an efficient electrocatalyst for the hydrogen evolution reaction in an alkaline electrolyte. *Sustainable Energy & Fuels* **2021**, *5*, 1059-1066.
5. Yin, H.; Zhao, S.; Zhao, K.; Muqsit, A.; Tang, H.; Chang, L.; Zhao, H.; Gao, Y.; Tang, Z. Ultrathin platinum nanowires grown on single-layered nickel hydroxide with high hydrogen evolution activity. *Nat. Commun.* **2015**, *6*, 6430.
6. Cao, Z.; Chen, Q.; Zhang, J.; Li, H.; Jiang, Y.; Shen, S.; Fu, G.; Lu, B.-a.; Xie, Z.; Zheng, L. Platinum-nickel alloy excavated nano-multipods with hexagonal close-packed structure and superior activity towards hydrogen evolution reaction. *Nat. Commun.* **2017**, *8*, 15131.
